# Further experimental results on modelling and algebraic control of a delayed looped heating-cooling process under uncertainties

**DOI:** 10.1016/j.heliyon.2023.e18445

**Published:** 2023-07-25

**Authors:** Libor Pekař, Radek Matušů, Petr Dostálek, Mengjie Song

**Affiliations:** a Faculty of Applied Informatics, Tomas Bata University in Zlín, Nad Stráněmi 4511, 76005, Zlín, Czech Republic; b Department of Technical Studies, College of Polytechnics Jihlava, Tolstého 1556, 58601, Jihlava, Czech Republic; c School of Mechanical Engineering, Beijing Institute of Technology, No. 5, Zhongguancun South Street, Haidian District, Beijing, 100081, China

**Keywords:** Algebraic control design, Long delays, Looped heating-cooling process, Robustness, Smith predictor, Uncertainties

## Abstract

The aim of this research is to revise and substantially extend experimental modelling and control of a looped heating-cooling laboratory process with long input-output and internal delays under uncertainties. This research follows and extends the authors' recent results. As several significant improvements regarding robust modelling and control have been reached, the obtained results are provided with a link and comparison to the previous findings. First, an infinite-dimensional model based on mathematical-physical heat and mass transfer principles is developed. All important heat-fluid transport and control-signal delays are considered when assembling the model structure and relations of quantities. Model parameter values optimization based on the measurement data follows. When determining static model parameter values, all variations in steady-state measured data are taken into account simultaneously, which enhances previously obtained models. Values of dynamic model parameters and delays are further obtained by least mean square optimization. This innovative model is compared to two recently developed process models and to the best-fit model that ignores the measured variations. Controller structures are designed using algebraic tools for all four models. The designed controllers are robust in the sense of robust stability and performance. Both concepts are rigorously assessed, and the obtained conditions serve for controller parameter tuning. Two different control systems are assumed: the standard closed-loop feedback loop and the two-feedback-controllers control system. Numerous experimental measurements for nominal conditions and selected perturbations are performed. Obtained results are further analyzed via several criteria on manipulated input and controlled temperature. The designed controllers are compared to the Smith predictor structure that is well-established for time-delay systems control. An essential drawback of the predictor regarding disturbance rejection is highlighted.

## 1 Introduction

Heating-cooling loops equipped with a heating power source and a heat sink connected by pipes appear typically in engines used in fuel-based power plants [1,2], and in cooling systems of an automotive combustion engine [3,4]. Their complete structures adopt a wide range of possible topologies [5]. The heat source and sink components can generally be viewed as heat-exchangers (HXs). Numerous academic research, experimental and industrial applications of processes with HXs and their networks have been investigated [6,7], which indicates that these problems attract a broad academic and engineering community.

Modelling and parameter identification of heating-cooling loops represent challenging research tasks. This fact is mainly due to process nonlinearities, signal constraints, time-varying parameters, and also uncertainties. The distributed nature of HXs and their loops brings about a necessity for advanced ad-hoc modelling strategies. To name just a few, a combination of distributed-parameter HX modelling and a spatial orthogonal-collocation discretization [8], a one-dimensional discretization of heat transfer balance equations [9], the conservation of mass and energy principle improved by log-mean-temperature-difference approach [10], or simplifying the convective-diffusion equations of the fluid flow [11].

The distributed nature of HXs together with fluid-flow latencies in pipelines imply the inevitable existence of the delay phenomenon and latencies in the process. Partial differential equations (PDEs) are widely used for HX system modelling based on the transport phenomenon mathematical description combined with the heat exchange between fluid and wall [5]. Their solutions include time-shifted quantities for particular HXs implicitly. However, many HX models in literature do not consider this non-simultaneous effect of quantities when the fluid is transported in pipelines. However, these delays can be very long and represent the most significant process latencies. For instance, the thermal behavior and importance of fluid flow through long pipelines were studied for district heating grids [1] or catalysts in diesel engines [12], yet fluid-flow delays are not considered explicitly.

Ordinary differential equations (ODEs), taking into account a variable time delay between the inlet and the outlet temperatures, were used for the modelling of solar thermal plants in Ref. [13]. A flow-dependent delay caused by the inlet temperature sensor position and the temperature-flow delay were considered in the model of a solar desalination plant [14]. When modelling a pipe network in a heating system with heating substations, pipe network thermal delay and building thermal inertia delay were determined in Ref. [15]. The cross-correlation function was used to estimate the delay values therein. The Hammerstein–Wiener model and a linear output-error model for a liquid–liquid HX system were identified in Ref. [16]. A delayed nonlinear parametric-uncertainty model of an HX used for pre-heating petroleum via hot water was applied in Ref. [17].

These examples motivated by combining ODEs obtained by the application of physical laws and mass transport latencies, however, have not considered time-shifted arguments in state or output variables. As looped heating-cooling processes include internal feedbacks, internal delays must also be taken into account next to the input-output ones. Besides the time-shifted quantities expressing lumped delays, convolution integrals can be utilized for distributed delays. The solution of these models has infinitely many modes. Hence, the models are infinite-dimensional. Although the so-called delay differential equations (DDEs) can be traced almost a century back (Volterra [18] already used past states when modelling predator-prey systems) their use in thermal and heating processes became attractive to researchers in the 1980s [19,20]. This concept was extended and elaborated more intensively, e.g., by Zhang and Nelson [21], who studied the delay effect on a building variable-air-volume ventilating system. Pipe and fluid temperature variations due to flow were analyzed in Ref. [22]. The so-called anisochronic modelling principle [23] was utilized when modelling a simple HX network. The principle is based on assuming all important process latencies and delays due to energy or fluid-flow (mass) transfer. Thermal systems with long ducts with a delay due to the fluid travelling time along the duct were analyzed in Ref. [24]. A general solution to the problem with a single duct and time-dependent ambient and inlet temperatures was obtained. The advantages of DDE models, ODE, and PDE approaches were discussed and demonstrated on a real solar heating system in Ref. [25]. A DDE model of a simple looped heating-cooling system equipped with an electric instantaneous water heater and a recovery waste-heat HX (a radiator plus fans) was designed in detail in Ref. [26]. Therein, the anisochronic modelling approach has been utilized for every single functional part of the appliance. The parts are interconnected via pipes yielding long input-output and state delays. Model parameters were identified in a two-step procedure, where parameters affecting the static gain were determined first, followed by the estimation of the rest. A relay-based identification strategy incorporating the so-called dominant spectrum subset of the model was proposed for the same appliance and model structure in Ref. [27]. A similar modelling idea was applied to a looped experimental heat transfer setup followed by a Krylov-based model order reduction procedure [28]. A very close appliance model was also used in Ref. [29].

It is worth noting that a novel modelling approach combining PDEs and DDEs into the so-called delay partial differential equations (DPDEs) was proposed for constant [30] and time-varying [5,31] flows recently. It comes from a one-dimensional PDE model that includes the transport process and the wall dynamics revealing delays.

Various control strategies adopting different design methods and tools for looped heating-cooling processes have been investigated during the last decades; however, most of them ignored delays in the model or control law design. For instance, the standard proportional-integral-derivative (PID) approach was compared to an advanced fuzzy-logic controller for a small heat exchanger network in Ref. [32], an adaptive dynamic matrix controller of heat sources constituting as a part of heat distribution systems was designed and implemented using a programmable logic controller in Ref. [33]. Thermal management control systems used in the automotive industry often neglect delays in the piping. For instance, a nonlinear model predictive controller [34] or a Lyapunov-based nonlinear controller [35] were derived for this purpose.

On the contrary, in large engine cooling loops, district heating, or HX networks, the dynamics is significantly influenced by the piping delays [5,36]. A controller based on a delayed infinite-dimensional model of an HX network was designed in Ref. [23]. Therein, controller parameters were tuned according to the desired dominant spectrum subset of the feedback control system. An HX system was controlled using the model predictive paradigm applied to a higher-order input-output-delay model combined with an artificial neural network in Ref. [17]. A fixed-order controller for a reduced-order delayed model of a looped HX laboratory process was derived and tested in Ref. [28]. The controller was tuned via the solution of a specific *H*_2_-norm optimization problem. Bušek et al. [29] proposed a functional-observer state-feedback proportional-integral (PI) controller set by the application of the Ackermann formula, equipped with a delayed anti-windup compensator. A laboratory heat transfer set-up model with long delays was controlled therein. Model-predictive self-tuning control design of this process was presented in Ref. [37]. The authors used a finite-dimensional model with input-output delays. The same appliance was also analyzed by different tools in Ref. [26]. The so-called fine temperature control of a heating-stations pipe network system to reduce energy consumption and carbon emission was proposed in Ref. [15]. Pipe network and building inertia thermal delays were included in the design.

In a recent work [38], an adaptive energy optimization control technique with a disturbance estimator, an energy consumption optimization block, a state predictor, and a state tracking controller for an advanced engine thermal management system has been proposed. The authors have pointed out that some control strategies [39,40] for looped coolant systems with a long dead time cause a large overshoot in temperature control. However, the internal delays were not taken into consideration. Moreover, the presented experiment has resulted in a non-zero reference-tracking steady-state error.

The robust control framework attempts to design and tune a control system sufficiently insensitively to process perturbations, model uncertainties, and signal disturbances. Especially, control system stability and a desired performance level should be satisfied. Besides advanced and computationally demanding robust control strategies applied to looped heating-cooling systems without considering delays in the design explicitly [11,41,42], other methods and results incorporating delayed models have been published. A robust temperature controller of a fluid-fluid HX process with uncertainty estimation was proposed in Ref. [43]. Therein, a delay induced by the actuator dynamics was considered in simulation tests. Santos et al. [14] designed a model predictive controller based on a quadratic program solution for a nonlinear solar collector model, where a robust input-output delay compensation scheme was proposed. A combination of the PI controller and the Clegg integrator compensator in the two-step robust temperature control under uncertainties and dominant time delay effect on the reset action was investigated in Ref. [44]. The controlled experimental HX system used in the food industry was modelled by a set of first-order plus time delay submodels. Using the same dead-time submodel, six robust controllers in real-time controlling a laboratory HX with nonlinear and asymmetric dynamics and with process gain, time-constant, and time-delay uncertainties were compared in Ref. [45]. The second-order plus time delay model of an HX process was used for robust controller design, ensuring stable control performance with model uncertainties in Ref. [46]. The controllers were tuned by the celebrated Ziegler-Nichols rules and by H-infinity synthesis. The third-order plus time delay model of a shell-and-tube HX was applied to derive, verify, and compare several robust controllers in Ref. [47]. The controllers were benchmarked using the integral absolute error (IAE) criterion. A model of a plate HX with a nonlinear static characteristic and time delay was utilized when performing a robust fuzzy evolving cloud-based control design [48]. Gupta et al. [16] considered different uncertainties (including that in the input-output delay value) in a linear model of a liquid-liquid HX process when deriving parameters of the conventional PID controller using the *H*_∞_ robustness metric.

It is worth noting that input-output delay models only (i.e., neither DDEs nor DPDEs) were considered when performing the above-given research. Results on advanced control of looped HX processes incorporating delayed infinite-dimensional models [5,[28], [29], [30]], however, have not utilized robustness methods and tools in the design explicitly. Moreover, they are mostly mathematically and computationally demanding. Recall that infinite dimensionality is natural for HXs and their loops; the former is due to an infinite-dimensional solution of PDEs, the latter because of the delay effect of long piping.

In [39], a control design method based on using algebraic tools [49] in the input-output space satisfying robust stability and performance was proposed when controlling the fluid temperature in a laboratory looped heating-cooling process. A Two-Feedback-Controllers (TFC) control system structure was used therein. A nonlinear process model derived based on DDEs was further linearized in the vicinity of a steady-state operating point for control design aims. Note that the results have been slightly refined in Ref. [50] later. The model and its parameters were obtained using a three-step procedure [26]. The second step estimated static parameters for each single process submodel separately and the results of each substep were used to determine the parameters of the following submodel. Another approach to identifying the model parameters based on the relay-feedback experiment was published in Ref. [27]. While the computational and experimental burden was less than in the previous method, worse results were obtained. However, the authors hypothesized that despite a low accuracy, the eventual model could be sufficient for control purposes.

Hence, the presented research has been motivated by gaps in the modelling and control design in the above-mentioned results and research questions raised therein. Moreover, the research is driven by a constant effort to develop advanced control techniques to reduce energy dependence while maintaining sufficient user comfort.

The motivation can be summarized as follows:

a) Using the mathematical-physical modelling via DDEs, the complete set of static parameters can be identified at once employing the least-square technique, i.e., not for each submodel separately. The obtained model should better cover and interpolate all the measurement variations and uncertainties than the original model [26], which adopted a part-by-part procedure.
b) Another two process model parameter sets are considered for the control design. First, it is a model that best estimates the nominal (unperturbed) process response. Such a model might perform worse robustness despite its better nominal accuracy. Second, the best model obtained by the relay-feedback test [27] is assumed. The research question is whether such a model can yield sufficient control responses.
c) The use of the standard simple One-Degree-of-Freedom (1DoF) control system can be compared to the TFC structure. The goal is to track the step-wise and linear-wise reference temperature value and asymptotically reject the constant load disturbance using algebraic tools.
d) In Ref. [39], the robust performance (that also includes robust stability) was not evaluated in detail. Therefore, the reader should be provided with detailed evaluations of all four models.
e) Both the control systems combined with all four models are benchmarked when real laboratory measurements on the appliance. The nominal case and three perturbed cases are considered.
f) Control target of engine cooling loops is to reach desired temperature levels while it is to reduce heat consumption in HX networks [5]. Hence, we consider both criteria when evaluating the experimental results.
g) As the process model structure includes input-output delay, the thing to ask is to use the well-established Smith predictor dead-time compensator [51] as an alternative to 1DoF and TFC. Hence, the Smith predictor controller and its properties are derived.

The rest of the paper is organized as follows. Section 2 concisely introduces the experimental laboratory appliance and its mathematical model via DDEs and auxiliary algebraic relations. Optimized robust model parameters identification is presented in Section 3. In addition, the unperturbed best-fit model, the original model [26], and the relay-based model [27] are given to the reader in the section as well. Controller structures via algebraic means for 1DoF and TFC control systems are derived in Section 4. Section 5 provides robust stability and performance conditions and their application to the derived models and control structures, giving rise to controller parameter settings. Real laboratory experiment results are displayed and evaluated by several performance measures in Section 6. Section 7 concisely links to the Smith dead-time compensator and highlights its drawback. Then the paper is concluded, and possible future research is sketched.

Note that only necessary information is placed in the main text body to be concise. Previously published data and extensive mathematical derivations and proofs are given in appendices, or the reader is referred to the literature. The basic used notation is summarized in a table.

## 2 Laboratory appliance and process model structure

A concise description of the looped heating-cooling process and the used laboratory test bed follows. A process model based on DDEs arising from heat balances and some auxiliary relations is also given to the reader [26,39].

### 2.1 Looped heating-cooling process description

**Fig. 1:**
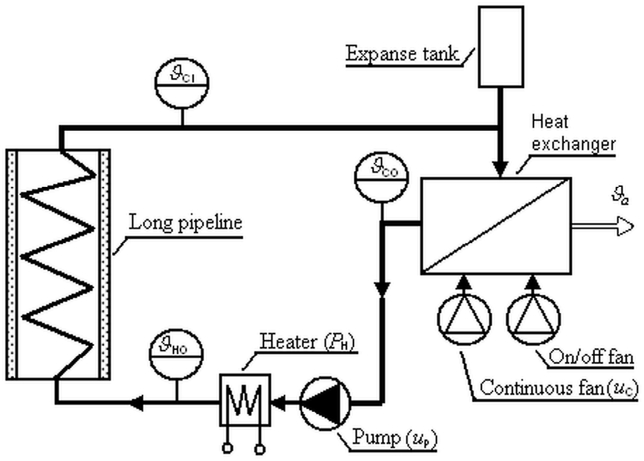
Looped heating-cooling process [39].

The looped heating-cooling process is sketched in Fig. 1 [39].

The process works as follows [26]: The heating fluid (distilled water here) in the loop is driven by a centrifugal pump controlled by the input voltage $u_{P}\left(t\right)$. It yields a change in the fluid flow rate $\dot{m}\left(t\right)$ in the piping. The fluid flows through an instantaneous water heater, the input power of which is $P_{H}\left(t\right)$. The heater outlet temperature of the fluid is $\vartheta_{\text{HO}}\left(t\right)$. The fluid then goes via a long well-insulated pipeline into a solid-liquid plate-and-fin HX (i.e., a radiator) that serves as a heat sink (let us call it a “cooler” for simplicity). The cooler fluid inlet temperature is measured as $\vartheta_{\text{CI}}\left(t\right)$. The rate of heat flow depends on the ambient temperature $\vartheta_{a}\left(t\right)$. The cooler fluid outlet temperature $\vartheta_{\text{CO}}\left(t\right)$ is affected mainly by the input voltage $u_{C}\left(t\right)$ of a fan connected to the cooler. The voltage value can be controlled continuously, whereas the second fan can only switch on or off. Changes in the heat fluid volume are compensated by a small expanse tank placed close to the cooler. Finally, the fluid flows into the pump, which closes the loop.

### 2.2 Laboratory appliance appearance and equipment

**Fig. 2:**
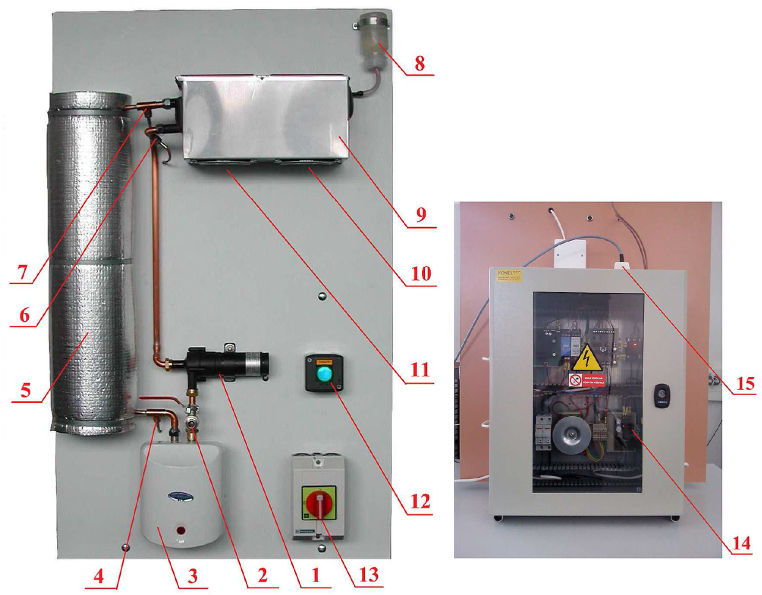
Laboratory appliance with the looped heating-cooling process [26].

The front and back sides of the laboratory appliance realizing the looped heating-cooling process are displayed in Fig. 2. Its technical description follows as per the positions indicated in the figure. Note that the reader is referred to Ref. [26] for further details about electronics inside the laboratory model, wiring, connection to a PC, and HW and SW equipment on the PC side.

Position 1 A magnetic drive centrifugal pump CM30P7-1 by Johnson continuously controlled by the input voltage within the range $u_{P}\left(t\right)\in \left[0,10\right]$ V.
Position 2 The inlet/outlet valve.
Position 3 The electric instantaneous water heater continuously controlled with the power range of $P_{H}\left(t\right)\in \left[0,750\right]$ W.
Position 4 A platinum resistance thermometer Pt1000 by Regmet returning the temperature value at the heater outlet calibrated to the range $\vartheta_{\text{HO}}\left(t\right)\in \left[0,100\right]$. The obtained value is stored and processed with a 14-bit resolution.
Position 5 The 15 m long insulated coiled copper pipeline.
Positions 6 and 7: Pt1000 thermometers giving cooler outlet and inlet temperatures $\vartheta_{\text{CO}}\left(t\right)$ and $\vartheta_{\text{CI}}\left(t\right)$, respectively.
Position 8 The expansion tank.
Position 9 The cooler (radiator) where fluid and gas phases interact.
Position 10 The on/off fan controlled by the transistor-transistor logic (TTL) signal.
Position 11 The fan continuously controlled by the input voltage $u_{C}\left(t\right)\in \left[0,10\right]$ V.
Position 12 The power on/off green-light indicator.
Position 13 The main switch.
Position 14 The main switchboard containing a microcontroller, resistance-to-voltage measurement converters, circuit breakers, semiconductor relays, and direct current power supplies.
Position 15 The 25-pin CANON 25 M connector.

### 2.3 DDEs-based process model structure

The modelling procedure based on the deductive principle has three steps [26]:

Step 1 Every distinct functional part of the process is modelled separately using DDEs based on heat transfer balances and via auxiliary algebraic equations expressing static relations of particular quantities. The obtained submodels are then linked via their mutual physical quantities due to fluid flow in the piping, which gives rise to delays. The crucial idea here is that all significant delays are considered.

Step 2 Heat transmission coefficients, variables in algebraic relations, and the mass flow rate are identified by steady-state input-output measurements, i.e., all derivatives in DDEs are assumed to be zero.

Step 3 Masses and delays are determined via measured dynamic responses by minimizing integral criteria in the time domain.

The following equations in this subsection summarize Step 1, whereas, Steps 2 and 3 are solved anew in Section 3 to improve the model robustness. Note that the following assumptions are taken into account when modelling: water inside the pipelines and functional parts of the process is incompressible; the fluid flow $\dot{m}\left(t\right)$ is constant in any place of the process within a particular time instant; the specific heat capacity c (≈4175 J kg^−1^ K^−1^) of the fluid is constant within a range of operating temperatures; the specific heat capacity of the piping wall material (copper) is neglected; the dynamics of sensors and actuators is omitted (see also Remark 1).

The heater dynamics is given by the imbalance between the inlet and outlet fluid heat, the input heat power from the solid surface of the heater, and the waste heat leaking through the heater shroud to the ambient environment. The particular DDE reads

(1) $$cM_{H}\frac{d\vartheta_{\text{HO}}\left(t\right)}{dt}=c\dot{m}\left(t\right)\left[\vartheta_{\text{HI}}\left(t-\tau_{H}\right)-\vartheta_{\text{HO}}\left(t\right)\right]+P_{H}\left(t-0.5\tau_{H}\right)-k_{H}\left(t\right)\left[\frac{\vartheta_{\text{HO}}\left(t\right)+\vartheta_{\text{HI}}\left(t-\tau_{H}\right)}{2}-\vartheta_{a}\left(t\right)\right]$$

where $M_{H}$ means the water mass inside the heater and $k_{H}\left(t\right)$ stands for the heat transmission coefficient, the value of which is assumed to be dependent on $P_{H}\left(t\right)$ and $\dot{m}\left(t\right)$ as

(2) kH(t)=hPH20(t)+h1m˙2(t)+h2PH(t)m˙(t)+h3h4PH(t)+h5m˙(t)

where $h_{i},i=\bar{0,5}$ are real-valued constants.

The duration of the heat fluid flow through the heater is $\tau_{H}$. Hence, the delay value $0.5\tau_{H}$ in (1) expresses that the heat power acts in the middle of the heater.

The long pipeline between the heater and the cooler can be modelled based on the heat balance and asynchronous effect of fluid temperatures on the pipeline inlet and outlet as

(3) $$cM_{P}\frac{d\vartheta_{\text{CI}}\left(t\right)}{dt}=c\dot{m}\left(t\right)\left[\vartheta_{\text{HO}}\left(t-\tau_{\text{HC}}\right)-\vartheta_{\text{CI}}\left(t\right)\right]-k_{P}\left[\frac{\vartheta_{\text{CI}}\left(t\right)+\vartheta_{\text{HO}}\left(t-\tau_{\text{HC}}\right)}{2}-\vartheta_{a}\left(t\right)\right]$$

where $M_{P}$ means the water mass inside the long pipeline, $k_{P}$ denotes the heat transmission coefficient (that is considered being a constant because of its low value), and $\tau_{\text{HC}}$ means the time during which the fluid goes from the heater outlet to the cooler inlet. Note that the arithmetical mean temperature on the right-hand side of (3) agrees with the case that the heat loss is constant along the pipeline at a time instant [26].

The cooler dynamics is modelled by a DDE analogously to (1) and (3) as

(4) $$cM_{C}\frac{d\vartheta_{\text{CO}}\left(t\right)}{dt}=c\dot{m}\left(t\right)\left[\vartheta_{\text{CI}}\left(t-\tau_{C}\right)-\vartheta_{\text{CO}}\left(t\right)\right]-k_{C}\left(t\right)\left[\frac{\vartheta_{\text{CO}}\left(t\right)+\vartheta_{\text{CI}}\left(t-\tau_{C}\right)}{2}-\vartheta_{a}\left(t\right)\right]$$

where $M_{C}$ means the water mass inside the cooler plates and tubes, $\tau_{C}$ denotes the fluid-flow transport delay between the cooler inlet and outlet, and $k_{C}\left(t\right)$ stands for the heat transmission coefficient, the value of which is assumed to be dependent on delayed control voltage $u_{C}\left(t\right)$.

(5) $$k_{C}\left(t\right)=c_{2}u_{C}^{2}\left(t-\tau_{\text{FC}}\right)+c_{1}u_{C}\left(t-\tau_{\text{FC}}\right)+c_{0}$$

where $\tau_{\text{FC}}$ is the fan reaction delay taking into account latencies of electronic and mechanical fan parts, and $c_{i},i=\bar{0,2}$ are real-valued constants. Note that the on/off fan (that influences $k_{C}\left(t\right)$) is permanently switched on.

The mass flow rate is related to $u_{P}\left(t\right)$ as follows

(6) $$\dot{m}\left(t\right)=\pi_{0}{\left[u_{P}\left(t\right)+\pi_{1}\right]}^{\pi_{2}}$$

in the model where $\pi_{i},i=\bar{0,2}$ are real-valued constants.

The fluid-flow delay $\tau_{\text{CH}}$ between the cooler outlet and the heater inlet (going through the pump) is modelled via a static relation only because of a short distance

(7) $$\vartheta_{\text{HI}}\left(t\right)=\vartheta_{\text{CO}}\left(t-\tau_{\text{CH}}\right)$$

that means that heat loss is omitted.

**Fig. 3:**
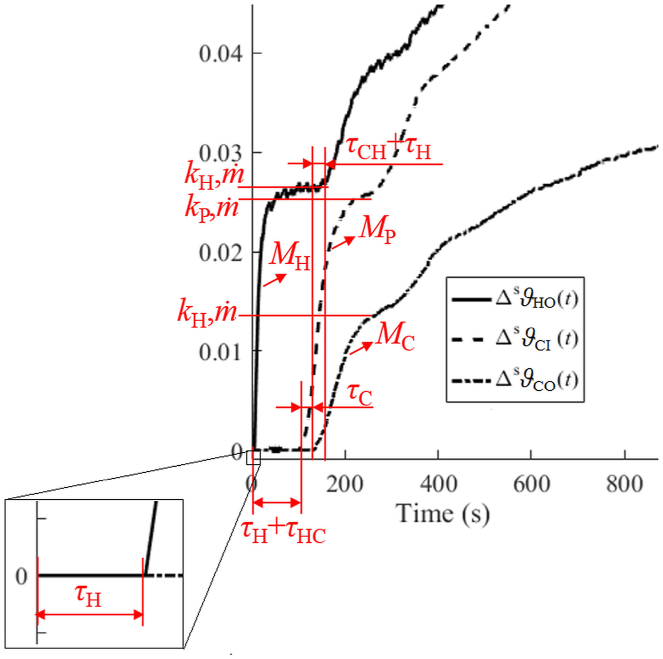
The effect of model variables, quantities, and delays on process unit step responses.

Fig. 3 displays the effect of particular model variables, quantities, and delays on process time-domain responses. Transfer part shapes of unit step responses on $\Delta P_{H}\left(t\right)$ (close to steady states ϑ·s) are taken as representatives in the figure.

Static gains (i.e., also steady-state temperature values) are affected by $\dot{m}\left(t\right),\vartheta_{a}\left(t\right),k_{H}\left(t\right),k_{P},k_{C}\left(t\right)$.

The following notation is used in Fig. 1:

(8) Δϑ·s(t):=ϑ·(t)−ϑ·s

Remark 1 Sensor delays are not considered in the model for several reasons, most of which yield from the fact that model delays are estimated based on the measurement, not derived analytically. First, as can be deduced from Fig. 1, almost all delays (except for $\tau_{H}$ and $\tau_{\text{FC}}$) are relative to time instants when significant shape changes of the characteristics appear. It is sufficient to detect the changes, not the temperature values themselves, for the delay values identification. Hence, these changes do not depend on sensors' time constants (≈8 s) that are much less than the overall internal delay (≈140 s) due to the loop closeness. In addition, possible sensor latencies have only a minor effect on the overall dynamics since they act in input-output relations only [27]. Last but not least, correct values of dead times are not crucial when experimental controlling the process because both delay-evaluation characteristics and controller inputs adopt the same measurement data.

## 3 Model parameters identification

The model parameters identification (see Steps 2 and 3 introduced in Subsection 2.3) is thoroughly revised and reformulated in this section, compared to the original result given in Ref. [26]. Recall that parameter values of every single functional part of the process were estimated separately therein. Now, let us perform the innovative concept that considers model (1), (2), (3), (4), (5), (6), and (7) at once.

### 3.1 Static parameters estimation

Let us call “static” those model parameters that affect the static gain. They remain in (1), (3), (4) when derivatives vanish after substituting algebraic relations (2), (5), (6), and the delay shift (7).

Consider the following ranges of inputs

(9) $$u_{P}\left(t\right)\in \left[4,6\right],u_{C}\left(t\right)\in \left[1,6\right],P_{H}\left(t\right)=\left[225,600\right]$$

The range of $u_{P}$ has been chosen relatively narrow for two reasons. First, the proposed control strategy is valid for constant delays; therefore, the flow rate induced by the pump input voltage should not significantly vary, and it ought to stay near the operating point. Second, $u_{P}$ does not pose to be the manipulated input. A relatively wide range of $u_{C}$ can serve to verify the robustness properties of the proposed control system by performing perturbations. The range of $P_{H}$ values is also wide as it serves as the manipulated input for controlling particular fluid temperatures. Besides, these heating power values are not symmetric with respect to the operating point but are shifted up. This feature is because of the assumption that heating rather than cooling is required for the process when controlling fluid temperature. It is also worth noting that the static characteristics for slightly wider ranges of $u_{P},u_{C}$, and $P_{H}$ can be estimated using linear extrapolation.

Steady values of fluid temperatures have been measured for ranges (9), as summarized in Appendix A [26]. Note that the values are normalized to the nominal constant ambient temperature of $\vartheta_{a}=24$ °C.

Substitute (2), (5), and (6) into (1), (3), and (4). Set zero derivatives on the left-hand sides of DDEs and let all variables be at their steady states (i.e., the delays vanish as well). Then, the set of obtained three nonlinear algebraic equations can be expressed in a condensed form

(10) f(v,p)=0

where $0={\left(0,0,\ldots ,0\right)}^{T}$, vT=(uPsuC,s,PHs,ϑHOs,ϑCIs,ϑas), and $p^{T}=\left(h_{0},...,h_{5},\pi_{0},\pi_{1},\pi_{2},c_{0},c_{1},c_{2},k_{P}\right)$. Define by J(v,p) the 3 × 13 Jacobian of f(v,p) with respect to p, i.e., each expression on the left-hand side of (10) is subject to partial derivatives according to each element in p.

Each line of measured data in Table A1 constitutes one real-valued vector $v_{i},i=\bar{1,34}$. Now define $f\left(p\right):={\left[f\left(v,p\right)\right]}_{v=v_{i},i=\bar{1,34}}$ and $J\left(p\right):={\left[J\left(v,p\right)\right]}_{v=v_{i},i=\bar{1,34}}$. Hence, f(p) and J(p) have dimensions of 102 × 1 and 102 × 13, respectively.

**Table 1:** Computed static model parameters – best results and the original one.

Parameter	Result 1	Result 2	Original
$h_{0}$	−0.3543	−7.794 × 10^−2^	2.4455
$h_{1}$	0.4616	1.0002	−1.7 × 10^−3^
$h_{2}$	112916.09	1.9799	55940.00
$h_{3}$	−85189.85	1.8835	−99225.69
$h_{4}$	33.1947	−5.1285	486.788
$h_{5}$	−123.186	−0.9817	777.692
$\pi_{0}$	5.432 × 10^−3^	4.516 × 10^−3^	4.186 × 10^−3^
$\pi_{1}$	−3.7155	−1.6928	0.2593
$\pi_{2}$	3.220 × 10^−2^	0.2560	0.3755
$c_{0}$	8.9125	9.6322	12.4063
$c_{1}$	−3.003 × 10^−2^	−3.389 × 10^−2^	0.8335
$c_{2}$	0.2360	0.2560	0.1197
$k_{P}$	0.2515	0.2735	0.3525
${\left\|f\left(p^{*}\right)\right\|}_{2}$	253.20	282.33	350.28

Then, the Levenberg-Marquardt method [52] enables finding the solution of f(p)=0 iteratively via (11)

(11) pk+1=pk−[Γk+γkdiag(Γk)]−1JT(pk)f(pk),Γk=JT(pk)J(pk)

where *k* means the iterative step. The method represents a stochastic approximation technique that minimizes the *H*_2_ norm of f(p), i.e., it solves nonlinear least-square problem (10).

Since the solution p*=limk→∞pk significantly depends on the initial estimate p0 and the evolution of the damping factor γ>0, it might not be unique. Table 1 displays the two best solutions obtained numerically, including the original result [26] for comparison. We call the mathematical model (1) to (7) with parameter values in the rightmost row of Table 1 the “Original model” hereinafter.

As can be seen from the table, the approximation error measured by the *H*_2_ norm has been reduced compared to the Original model. Another advantage is that the results cover a broad spectrum of process perturbations and measurement uncertainties, which is beneficial for the further robust control design. On the other hand, it may lead to a worse estimation of the nominal case.

Nevertheless, Result 1 in Table 1 is unsuitable for robust control design since it enables only a reduced range of pump voltage input (i.e., a range of fluid flow values). Namely, it is clear from (6) that whenever $u_{P}\left(t\right)<-\pi_{1}$, the flow rate $\dot{m}\left(t\right)$ becomes complex-valued. That is, Result 1 admits $u_{P}\left(t\right)\geq 3.7155$ V, which is rather limiting (regardless, it complies with ranges (9)). Therefore, we have selected Result 2 in Table 1 for further identification and control in this research.

### 3.2 Dynamic parameters estimation

The so-called “dynamic” parameters influence the transient part of a time-domain response (see Fig. 3). These are masses $M_{H},M_{P},M_{C}$ and all delays $\tau_{H},\tau_{\text{HC}},\tau_{C},\tau_{\text{CH}},\tau_{\text{FC}}$. The error minimization between the measured and modelled responses can estimate their values. However, as the static parameters determined in the preceding subsection lead to incorrect static gain estimation (due to perturbations), this gain can be easily adjusted by an additional gain, so that the steady-state values coincide. Nevertheless, such an adjustment may harm other decisive parts of the responses indicated in Fig. 3. Therefore, the dynamic parameter values have eventually been found for Result 2 of Table 1 based on the following intuitive assumption:

Assumption 1 Consider a model that matches the nominal responses perfectly. Then, its dynamic parameter values must be optimal for the model under perturbations.

To rephrase Assumption 1, the complete model that fits the measured nominal measured data in the vicinity of some operating point optimally is found first. Let us denote this model simply as “Best-fit”. Then, the model partially found in Subsection 3.1 adopts the dynamic parameters of the best-fit one.

As the aim is to use $P_{H}\left(t\right)$ as the manipulated input for the control tasks, only responses (of temperatures) to the step change $\Delta P_{H}\left(t\right)$ are assumed when finding the best-fit model parameters. Let the objective be the IAE between the measured ($\vartheta_{\cdot }\left(t\right)$) and modelled ($\vartheta_{\cdot ,m}\left(t\right)$) responses

(12) $${\text{IAE}}_{\vartheta_{\cdot }}:=\int_{t_{0}}^{t_{1}}\left|\vartheta_{\cdot }\left(t\right)-\vartheta_{\cdot ,m}\left(t\right)\right|dt\approx \Delta t\sum_{k=t_{0}/\Delta t}^{t_{1}/\Delta t}\left|\vartheta_{\cdot }\left(k\right)-\vartheta_{\cdot ,m}\left(k\right)\right|$$

where Δt is the sampling time. Hence, the cost function of the optimization problem for the best-fit model can be defined as follows:

(13) $$\min\left({\text{IAE}}_{\vartheta_{\text{HO}}}+{\text{IAE}}_{\vartheta_{\text{CI}}}+{\text{IAE}}_{\vartheta_{\text{CO}}}\right)$$

Let us select the following operating point for the nominal data

(14) (uPs,uCs,PHs,ϑHOs,ϑCIs,ϑCOs,ϑas)=(5V,3V,300W,43.22°C,43.00°C,34.92°C,24°C)

The solution of (13) for $t_{0}=0\;s,t_{1}=2000\;s,\Delta t=1\;s$ using the well-established Nelder-Mead flexible-simplex method [53] yields the dynamic parameters provided in Table 2. The table also contains the values of the original model [26].

**Table 2:** Computed best-fit dynamic and original models’ parameters.

Parameter	Best-fit	Original
*M*_H_	9.338 × 10^−2^	8.109 × 10^−2^
*M*_P_	2.944 × 10^−2^	2.190 × 10^−1^
*M*_C_	2.239 × 10^−2^	2.717 × 10^−1^
$\tau_{H}$	4.61	3
$\tau_{\text{HC}}$	102.36	118
$\tau_{C}$	26.25	23
$\tau_{\text{CH}}$	8.07	7
$\tau_{\text{FC}}$	11.5	12

The value of $\tau_{\text{FC}}$ have been found graphically based on the response to ΔuCs(t). Note that other delays can alternatively be found based on Fig. 1. That is, the four particular time intervals can also be identified from the measured data, giving rise to $\tau_{H},\tau_{\text{HC}},\tau_{C},\tau_{\text{CH}}$.

Let us denote by “Model 1” the novel model having static parameters introduced as “Result 2” in Table 1 and dynamic parameters provided as “Best-fit” in Table 2.

The knowledge of nominal delay values and particular modelled relations $u_{P}\left(t\right)\mapsto \dot{m}\left(t\right)$ enables estimating the operating ranges (or possible perturbations) of the delays. Relations (15) express that delays are in inverse proportion to $\dot{m}\left(t\right)$.

(15) $$\tau_{\cdot ,\max}=\tau_{\cdot ,\text{nominal}}\frac{{\dot{m}}_{\min}}{{\dot{m}}_{\text{nominal}}},\tau_{\cdot ,\min}=\tau_{\cdot ,\text{nominal}}\frac{{\dot{m}}_{\max}}{{\dot{m}}_{\text{nominal}}}$$

The obtained ranges are summarized in Table 3.

**Table 3:** Computed ranges of delays for $u_{P}\left(t\right)\in \left[4,6\right]$.

Delay	Original	Model 1	Best-fit
$\tau_{H}$	[2.77, 3.20]	[4.25, 4.89]	[3.69, 5.53]
$\tau_{\text{HC}}$	[109.02, 125.97]	[94.37, 108.64]	[81.88, 122.83]
$\tau_{C}$	[21.25, 24.55]	[24.20, 27.86]	[21.00, 31.50]
$\tau_{\text{HC}}$	[6.47, 7.47]	[7.44, 8.57]	[6.46, 9.68]

### 3.3 Model linearization and results comparison

As the aim of this research is to apply robust control principles for linear systems, nonlinear model (1)–(7) has to be linearized. Appendix 2 follows a simple linearization procedure in the neighbourhood of the operating point (14) introduced in Ref. [26].

**Fig. 4:**
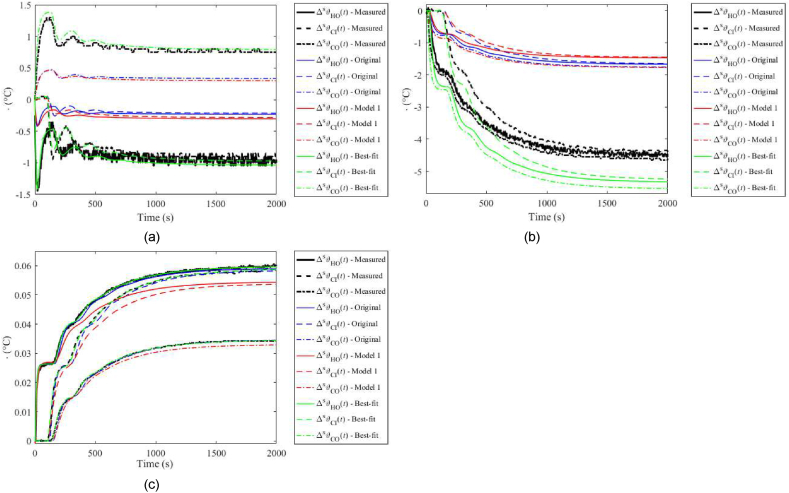
Unit step responses (without static gains adjustment) for ΔuPs(t) (a), ΔuCs(t) (b), and ΔPHs(t) (c).

**Fig. 5:**
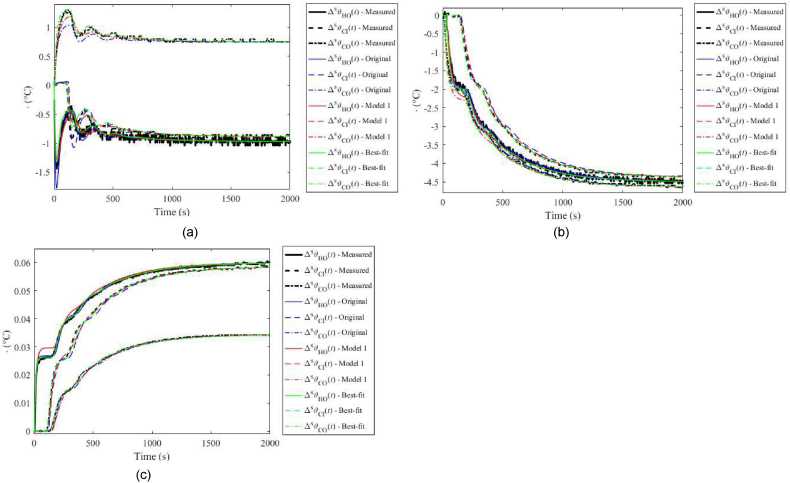
Unit step responses (with static gains adjustment) for ΔuPs(t) (a), ΔuCs(t) (b), and ΔPHs(t) (c).

A comparison of unit step responses for input and output variables is displayed in Fig. 4(a–c). Original model, Model 1, and the Best-fit model are included for the benchmark. Fig. 5 includes those unit step responses for the unified static gain. As mentioned above, the modelled responses can simply be adjusted using an auxiliary gain in practice.

The figures prove the Best-fit model superiority when matching the nominal step responses. Plots for the Original model and Model 1 in Fig. 4(a–c) evince significant errors when responding to ΔuPs(t) and ΔuCs(t). The main reason is that both models attempt to cover the perturbations and uncertainties, and the nominal case represents only a limited subset of the measured data. It is also partially due to the dynamic model parameters have been found based on the response to ΔPHs(t); however, transient parts of responses are estimated quite well, as clear from Fig. 5(a–c). Whereas the Original model works better in cases (b) and (c), Model 1 matches the measured responses better in case (a). It must, however, be highlighted again that the best matching of the nominal case is not the primary goal of these models.

In [27], various models of the looped heating-cooling process in question were received by a relay-feedback experiment. These relay-based models have been computed for relation $P_{H}\left(t\right)\mapsto \vartheta_{\text{CO}}\left(t\right)$ only. It can be deduced from (B.1) that the relation is given by the DDE

(16) Δϑ…s(t)+a2Δϑ¨s(t)+a1Δϑ˙s(t)+a0Δϑs(t)+a0DΔϑs(t−τa)=b0ΔPHs(t−τb)+b0DΔPHs(t−τb−τ0)

where $\tau_{0}=0.5\tau_{H},\tau_{a}=\tau_{H}+\tau_{\text{HC}}+\tau_{C}+\tau_{\text{CH}},\tau_{b}=\tau_{\text{HC}}+\tau_{C}$. Four particular models with the best integral measures in the time and frequency domains are provided in Table 4. Coefficients of (16) for the Original model, Model 1, and the Best-fit model are also included in the table for completeness. Note that this data will be used in the next sections. Surprisingly, although Model 1 adopts the dynamic parameters of the Best-fit model, the coefficients of the latter one are closer to the Original model.

**Table 4:** Computed nominal parameters of submodel (16).

Parameter	Relay 1	Relay 2	Relay 3	Relay 4	Original	Model 1	Best-fit
$a_{2}$	132.0240	1.5388	43.8899	4.4339	0.1722	0.1221	0.1783
$a_{1}$	169.0525	40237.93	23948.84	31243.89	8.510 × 10^−3^	4.325 × 10^−3^	9.412 × 10^−3^
$a_{0}$	3.060 × 10^−2^	−17.1592	−3.4535	−5.054 × 10^−4^	1.299 × 10^−4^	4.578 × 10^−5^	1.497 × 10^−4^
$a_{0D}$	0.3820	127.4072	92.1380	107.4560	−7.219 × 10^−5^	−2.308 × 10^−5^	−8.325 × 10^−5^
$b_{0}$	0.3070	78.6312	−30.4525	46.3542	−2.655 × 10^−7^	−2.821 × 10^−7^	−8.928 × 10^−10^
$b_{0D}$	−0.2935	−75.0398	33.3415	−42.8537	2.275 × 10^−6^	1.030 × 10^−6^	2.278 × 10^−6^
$\tau_{a}$	12.11	16.37	4.70	7.06	151	141.28	141.28
$\tau_{b}$	154.78	154.45	117.68	140.66	141	128.60	128.60
$\tau_{0}$	4.61	3.66	3.65	4.18	1.5	2.31	2.31

Let the best model (measured by the IAE) in the first column (i.e., Relay 1) of Table 4 be the nominal “Relay model” for control purposes. Notice that non-delay coefficients differ from other models significantly, and the internal delay $\tau_{a}$ is far from the physical one of the process. The reason for including the relay-based models is the following. This research question was raised in Ref. [27]: Can the process be sufficiently controlled based on the (relatively inaccurate) relay-based model? Hence, a partial goal of this research is to benchmark the model usability in the robust control design against other process models.

**Fig. 6:**
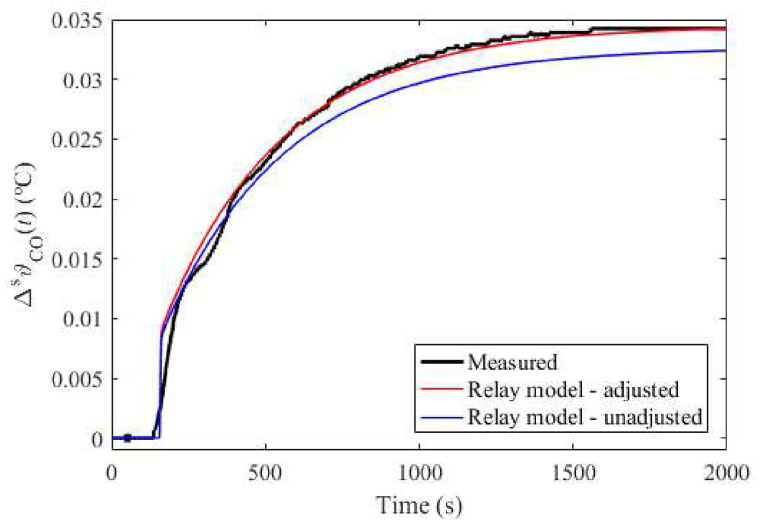
Unit step responses (with/without static gains adjustment) ΔPHs(t)↦ΔϑCOs(t) for the Relay model.

Unit step responses ΔPHs(t)↦ΔϑCOs(t) for the Relay model with/without the static gain adjustment are displayed in Fig. 6. Note that the IAEs (after the unifying of the static gains, i.e., the steady-state temperatures coincide) for the Original model, Model 1, Best-fit model, and Relay model are, respectively, 0.114, 0.098, 0.053, and 0.984.

**Fig. 7:**
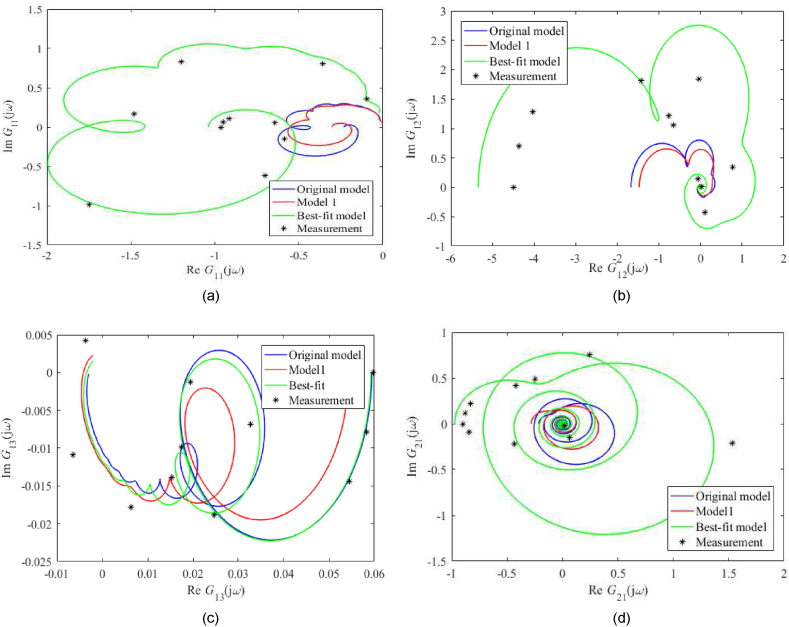

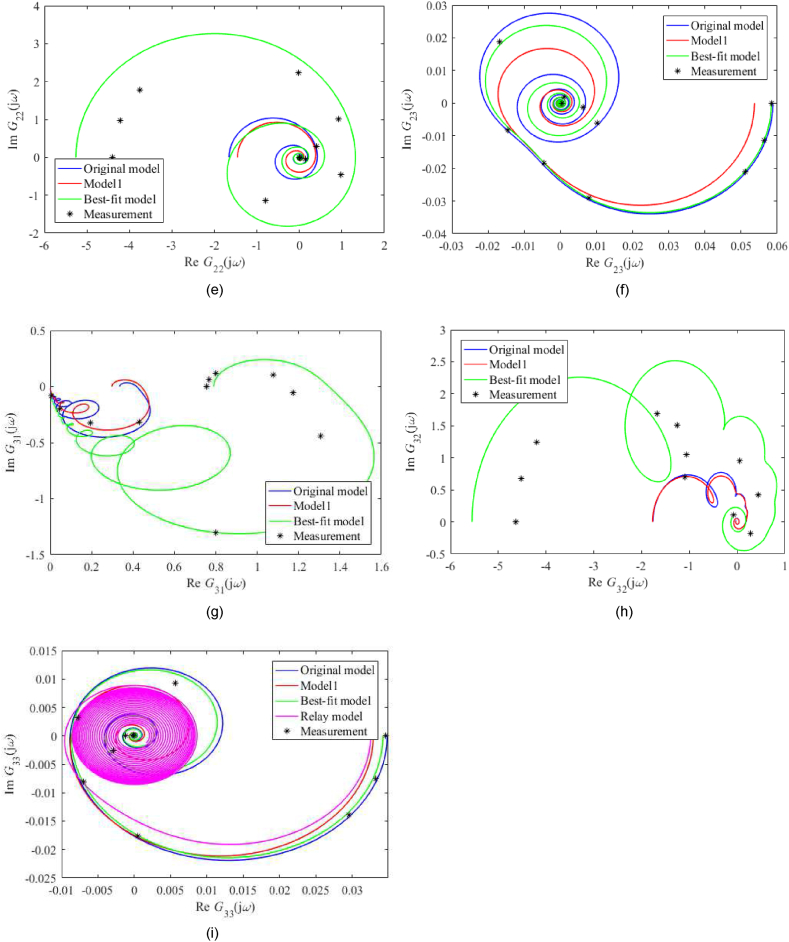
Nyquist plots (without static gains adjustment) of *G*_11_ (a), *G*_12_ (b), *G*_13_ (c), *G*_21_ (d), *G*_22_ (e), *G*_23_ (f), *G*_31_ (g), *G*_32_ (h), *G*_33_ (i).

**Fig. 8:**
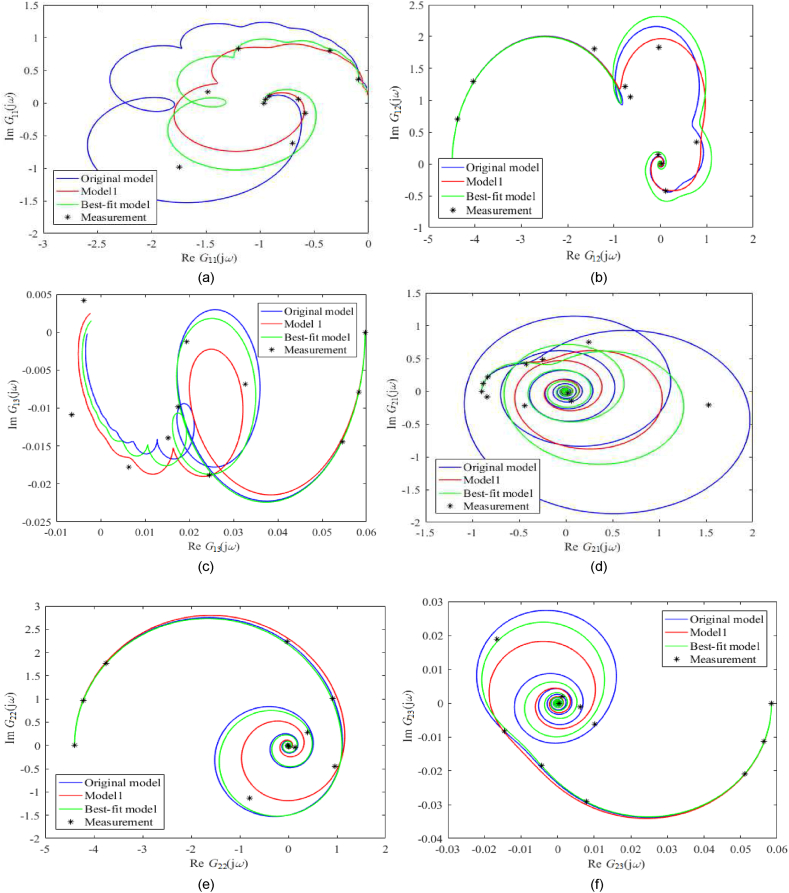

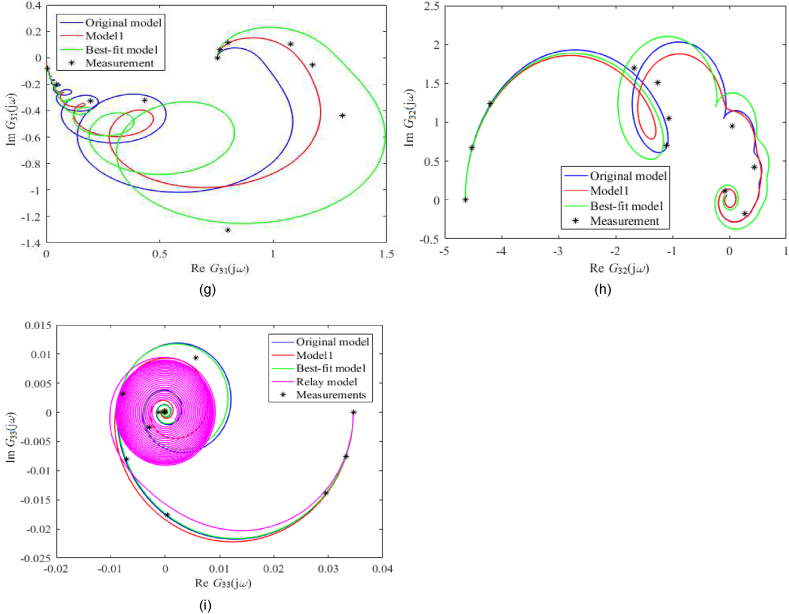
Nyquist plots (with static gains adjustment) of *G*_11_ (a), *G*_12_ (b), *G*_13_ (c), *G*_21_ (d), *G*_22_ (e), *G*_23_ (f), *G*_31_ (g), *G*_32_ (h), *G*_33_ (i).

A frequency-domain model comparison is made via Nyquist plots; see Fig. 7(a–i) for unadjusted static gain and Fig. 8(a–i) for adjusted ones. Note that the actual measured process data was obtained simply by inserting the sinus signal into the particular input (at the operation point) and observing the amplitude and phase shift, not via the Fourier series analysis. In the figures, $G_{ij}\left(s\right)$ means the entry of the transfer function matrix G(s) in (B.1).

Unadjusted nominal frequency responses for the Best-fit model naturally provide the best matching among all the models. Although the shapes of all plots are similar, their scales differ significantly, mainly due to process perturbations that have to be covered. Contrariwise, Nyquist plots with adjusted static gains are similar in shape and size in many cases. In Fig. 8(a–i), the Original model and Model 1 estimated the measured data well (besides the Best-fit model does).

## 4 Controller structure design by algebraic tools

In this section, the reader is provided with a concise description of the used control system structures, namely, 1DoF and TFC. Parameterized control laws are then derived for each structure using algebraic tools introduced in Appendix C.

The goal is to control $\vartheta_{\text{CO}}\left(t\right)$ using the manipulated input $P_{H}\left(t\right)$ based on submodel (16). Other external inputs, i.e., $u_{P}\left(t\right),u_{C}\left(t\right),\vartheta_{a}\left(t\right)$ are considered being model uncertainties. The transfer function of relation (16) reads

(17) $$G_{m}\left(s\right)=\frac{b_{0}+b_{0D}e^{-\tau_{0}s}}{s^{4}+a_{3}s^{3}+a_{2}s^{2}+a_{1}s+a_{0}+a_{0D}e^{-\tau_{a}s}}e^{-\tau_{b}s}=\frac{b\left(s\right)}{a\left(s\right)}$$

### 4.1 1DoF and TFC control systems

**Fig. 9:**
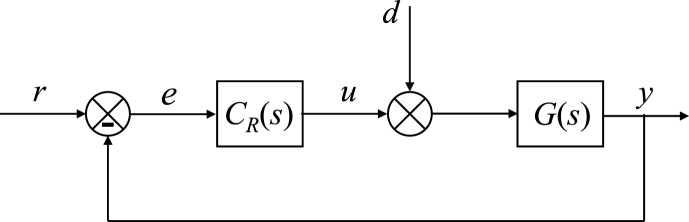
1DoF control system.

The 1DoF control system agrees with the simple negative control feedback loop; see Fig. 9.

In the figure, r(t),e(t),u(t),d(t),y(t) denote the reference, control error, control action (i.e., the computed manipulated input), load disturbance, and the output variables, respectively. The controlled system and controller transfer functions are $G\left(s\right),C_{R}\left(s\right)$, respectively.

**Fig. 10:**
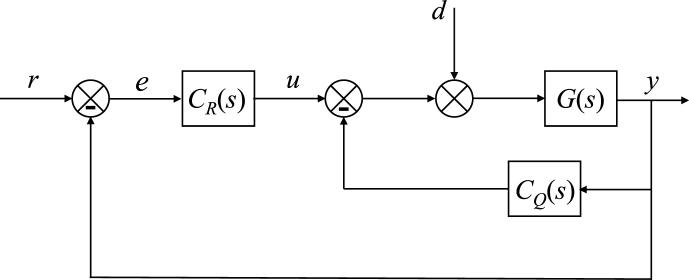
TFC control system.

The TFC scheme displayed in Fig. 10 includes an additional inner feedback loop that may help to stabilize the control system, adjust its dynamics or attenuate the disturbance effect by an additional degree of freedom. The transfer function of the controller acting inside the inner loop is denoted as $C_{Q}\left(s\right)$.

### 4.2 Algebraic representation of signals and transfer functions in the control systems

Algebraic control design in the ring of quasi-polynomial meromorphic functions ($R_{QM}$) is adopted [49,50]. The reader is referred to Appendix C for the ring definition (Definition C.4). The controller design idea is based on the fractional representation of all control system transfer functions and signals using $R_{QM}$. One can write

(18) $$\begin{array}{l} r\left(s\right)=\frac{H_{r}\left(s\right)}{F_{r}\left(s\right)},d\left(s\right)=\frac{H_{d}\left(s\right)}{F_{d}\left(s\right)},G\left(s\right)=\frac{B\left(s\right)}{A\left(s\right)},C_{R}\left(s\right)=\frac{R\left(s\right)}{P\left(s\right)},C_{Q}\left(s\right)=\frac{Q\left(s\right)}{P\left(s\right)}\text{,}\\ F_{r}\left(s\right),F_{D}\left(s\right),H_{r}\left(s\right),H_{d}\left(s\right),A\left(s\right),B\left(s\right),Q\left(s\right),P\left(s\right),R\left(s\right)\in R_{QM} \end{array}$$

The following three goals to be satisfied are as follows:

a) The control system is stable, i.e., all transfer functions are in $R_{QM}$.
b) The output asymptotically approaches the reference, i.e., $\lim_{t\to \infty }y\left(t\right)=\lim_{t\to \infty }r\left(t\right)$ or $\lim_{t\to \infty }e\left(t\right)=0$ for d(t)=0,r(t)≠0.
c) The load disturbance is asymptotically attenuated, i.e., $\lim_{t\to \infty }y\left(t\right)=0$ for d(t)≠0,r(t)=0.

### 4.3 Controller structure design for 1DoF

The following three lemmas can be proven [49].

Lemma 1 Let $A\left(s\right),B\left(s\right)\in R_{QM}$ in (18) be coprime. The 1DoF control system is stable if and only if there exists a coprime pair $R\left(s\right),P\left(s\right)\in R_{QM}$ satisfying

(19) A(s)P(s)+B(s)R(s)=1

Note that a particular solution pair $R_{p}\left(s\right),P_{p}\left(s\right)$ can further be parameterized as

(20) $$P\left(s\right)=P_{p}\left(s\right)\pm B\left(s\right)Z\left(s\right),R\left(s\right)=R_{p}\left(s\right)\mp A\left(s\right)Z\left(s\right)$$

for $P\left(s\right)\neq 0,Z\left(s\right)\in R_{MS}$.

Lemma 2 The reference is asymptotically tracked if and only if (21) holds

(21) $$F_{r}\left(s\right)\;\text{divides}\;A\left(s\right)P\left(s\right)$$

Lemma 3 The load disturbance is asymptotically rejected if and only if (22) holds

(22) $$F_{d}\left(s\right)\;\text{divides}\;B\left(s\right)P\left(s\right)$$

Now, consider controlled process model (17). A controller that satisfies all three above-given conditions for a linear-wise r(t) and a step-wise d(t) (that is a natural demand in practice) has the transfer function

(23) $$C_{R}\left(s\right)=\frac{m_{0}m_{1}a\left(s\right)\left(r_{1}s+r_{0}\right)}{p_{num}\left(s\right)}$$

where

(24) $$\begin{array}{l} r_{1}=2\left(m_{0}+m_{1}\right)\left(b_{0}+b_{0D}\right)+m_{0}m_{1}\left[b_{0}\tau_{b}+b_{0D}\left(\tau_{b}+\tau_{0}\right)\right],r_{0}=m_{0}m_{1}\left(b_{0}+b_{0D}\right)\text{,}\\ p_{num}\left(s\right)=p_{num,4}s^{4}+p_{num,3}s^{3}+p_{num,2}s^{2}+p_{num,1}\left(s\right)s+p_{num,0}\left(s\right),p_{num,4}={\left(b_{0}+b_{0D}\right)}^{2}\text{,}\\ p_{num,3}=2{\left(b_{0}+b_{0D}\right)}^{2}\left(m_{0}+m_{1}\right),p_{num,2}={\left(b_{0}+b_{0D}\right)}^{2}\left[m_{0}^{2}+4m_{0}m_{1}+m_{1}^{2}\right]\text{,}\\ p_{num,1}\left(s\right)=m_{0}m_{1}\left[2\left(m_{0}+m_{1}\right){\left(b_{0}+b_{0D}\right)}^{2}-r_{1}b\left(s\right)\right],p_{num,0}\left(s\right)=m_{0}m_{1}r_{0}\left[\left(b_{0}+b_{0D}\right)-b\left(s\right)\right] \end{array}$$

and $m_{0},m_{1}>0$ are selectable controller parameters. A detailed controller derivation is given in Appendix D.

### 4.4 Controller structure design for TFC

For the TFC structure, the following lemmas hold [39,50].

Lemma 4 Let $A\left(s\right),B\left(s\right)\in R_{QM}$ in (18) be coprime. The TFC control system is stable if and only if there exists a coprime pair $V\left(s\right),P\left(s\right)\in R_{QM}$ satisfying

(25) A(s)P(s)+B(s)V(s)=1

where V(s) can be written by (26)

(26) V(s)=R(s)+Q(s)

A particular solution $V_{p}\left(s\right),P_{p}\left(s\right)$ can further be parameterized as

(27) $$P\left(s\right)=P_{p}\left(s\right)\pm B\left(s\right)Z\left(s\right),V\left(s\right)=V_{p}\left(s\right)\mp A\left(s\right)Z\left(s\right)$$

for $P\left(s\right)\neq 0,Z\left(s\right)\in R_{MS}$.

Lemma 5 The reference is asymptotically tracked if and only if

(28) $$F_{r}\left(s\right)\;\text{divides}\;A\left(s\right)P\left(s\right)+B\left(s\right)Q\left(s\right)$$

Lemma 6 The load disturbance is asymptotically rejected if and only if

(29) $$F_{d}\left(s\right)\;\text{divides}\;B\left(s\right)P\left(s\right)$$

Consider a linear-wise r(t) and a step-wise d(t) again. Possible controllers satisfying control feedback stability, asymptotic reference tracking, and load disturbance rejection are governed by the transfer function

(30) $$C_{Q}\left(s\right)=\frac{m_{0}^{3}a\left(s\right)\lambda v_{1}s^{2}}{p_{num}\left(s\right)\left(s+m_{1}\right)},C_{R}\left(s\right)=\frac{m_{0}^{3}a\left(s\right)\left[\left(1-\lambda \right)v_{1}s^{2}+\left(v_{1}m_{1}+v_{0}\right)s+m_{1}v_{0}\right]}{p_{num}\left(s\right)\left(s+m_{1}\right)}$$

where

(31) $$\begin{array}{l} v_{1}=4\left(b_{0}+b_{0D}\right)+m_{0}\left[b_{0}\tau_{b}+b_{0D}\left(\tau_{b}+\tau_{0}\right)\right],v_{0}=m_{0}\left(b_{0}+b_{0D}\right)\text{,}\\ p_{num}\left(s\right)=p_{num,4}s^{4}+p_{num,3}s^{3}+p_{num,2}s^{2}+p_{num,1}\left(s\right)s+p_{num,0}\left(s\right),p_{num,4}={\left(b_{0}+b_{0D}\right)}^{2}\text{,}\\ p_{num,3}=4m_{0}{\left(b_{0}+b_{0D}\right)}^{2},p_{num,2}=6m_{0}^{2}{\left(b_{0}+b_{0D}\right)}^{2}\text{,}\\ p_{num,1}\left(s\right)=m_{0}^{3}\left[{\left(b_{0}+b_{0D}\right)}^{2}-r_{1}b\left(s\right)\right],p_{num,0}\left(s\right)=m_{0}^{3}r_{0}\left[\left(b_{0}+b_{0D}\right)-b\left(s\right)\right] \end{array}$$

and $m_{0},m_{1}>0,\lambda \in (0,1]$ are selectable controller parameters. Clearly, the TFC control system has one more tunable parameter than the 1DoF system (in our case). Moreover, reference tracking and disturbance rejection tasks can be partially solved independently. A detailed derivation of the controllers can be found in Appendix E.

## 5 Robust stability and performance

Based on models obtained in Sections 2, 3, parameters of controllers (23)–(24) and (30)–(31) are set to meet robustness requirements. Namely, robust stability and robust performance are considered [54]. Roughly speaking, when these requirements are met, the control feedback system remains stable and provides a satisfactory response under process perturbations and model uncertainties. The perturbations are due to all variations of uncontrolled inputs, see (9), and the fluctuation of the ambient temperature $\vartheta_{a}\in \left[18,28\right]$ °C. Recall that the nominal setting is given by the operating point (14).

The nominal model transfer function $G_{m,0}\left(s\right)$ have structure (17) with parameters given in Table 4. The set of all perturbed transfer functions $G_{m}\left(s\right)$ satisfies unstructured multiplicative uncertainties (32)

(32) $$G_{m}\left(s\right)=\left[1+\Delta \left(s\right)W_{M}\left(s\right)\right]G_{m,0}\left(s\right)$$

where ${\left\|\Delta \left(s\right)\right\|}_{\infty }\leq 1$ is a stable bounded function. $W_{M}\left(s\right)$ expresses a fixed stable weight function of the uncertainty frequency distribution and is searched so that inequality

(33) $$\left|\frac{G_{m}\left(j\omega \right)}{G_{m,0}\left(j\omega \right)}-1\right|\leq \left|W_{M}\left(j\omega \right)\right|,\forall \omega \geq 0$$

is satisfied without an excessive conservativeness.

Definition 1 The control system is robustly stable if it is stable for $G_{m,0}\left(s\right)$ and remains stable also for all $G_{m}\left(s\right)$.

Definition 2 The control system satisfies the nominal performance if it holds that

(34) $${\left\|S_{0}\left(j\omega \right)\right\|}_{\infty }\leq {\left\|W_{P}\left(j\omega \right)\right\|}_{\infty }^{-1}\Leftrightarrow \left|S_{0}\left(j\omega \right)\right|\leq {\left|W_{P}\left(j\omega \right)\right|}^{-1},\forall \omega \geq 0$$

where $W_{P}\left(s\right)$ is the sensitivity weight function and $S_{0}\left(s\right)$ stands for the nominal sensitivity function that agrees with the transfer function between r(t) and e(t) for the nominal model $G_{m,0}\left(s\right)$.

To rephrase Definition 2, inequality (34) expresses that the nominal control system performs “sufficiently well”, as per the selected bound $1/W_{P}\left(s\right)$.

Definition 3 The control system satisfies the robust performance if it is robustly stable and satisfies (34) for all perturbed transfer functions $G_{m}\left(s\right)$, i.e.,

(35) $${\left\|S\left(j\omega \right)\right\|}_{\infty }\leq {\left\|W_{P}\left(j\omega \right)\right\|}_{\infty }^{-1}$$

where S(s) means the sensitivity function for the whole set of $G_{m}\left(s\right)$.

Particular conditions for 1DoF and TFC control systems and their application to the heating-cooling process models and their derived controllers follow.

### 5.1 Robustness design for 1DoF

The following lemmas hold for the 1DoF control system under the assumption that $G_{m,0}\left(s\right)$ and $G_{m}\left(s\right)$ have the equal number of poles $s_{i}$ with $Res_{i}\geq 0$ [54].

Lemma 7 The control system is robustly stable if and only if

(36) $${\left\|RS_{1\text{DoF}}\left(j\omega \right)\right\|}_{\infty }:={\left\|W_{M}\left(j\omega \right)T_{0}\left(j\omega \right)\right\|}_{\infty }<1$$

where $T_{0}\left(s\right)=1-S_{0}\left(s\right)$ is the nominal complementary sensitivity function that agrees with the transfer function between r(t) and y(t) for the nominal model $G_{m,0}\left(s\right)$.

For 1DoF with controller (23)- (24), it holds that

(37) $$T_{0}\left(s\right)=\frac{C_{R}\left(s\right)G_{m,0}\left(s\right)}{1+C_{R}\left(s\right)G_{m,0}\left(s\right)}=B\left(s\right)R\left(s\right)=\frac{b\left(s\right)}{m\left(s\right)}\frac{m_{0}m_{1}\left(r_{1}s+r_{0}\right)}{\left(s+m_{0}\right){\left(b_{0}+b_{0D}\right)}^{2}}$$

with the nominal parameters in Table 4.

Lemma 8 The control system satisfies inequality (35) and robust stability condition (36) if and only if

(38) $$RP_{1\text{DoF}}:={\left\|RS_{1\text{DoF}}\left(j\omega \right)\right\|}_{\infty }+{\left\|S_{0}\left(j\omega \right)W_{P}\left(j\omega \right)\right\|}_{\infty }<1$$

Note that $S_{0}$ is given by (39)

(39) $$S_{0}\left(s\right)=1-T_{0}\left(s\right)=\frac{1}{1+C_{R}\left(s\right)G_{m,0}\left(s\right)}=A\left(s\right)P\left(s\right)=\frac{a\left(s\right)}{m\left(s\right)}\frac{p_{num}\left(s\right)}{{\left(b_{0}+b_{0D}\right)}^{2}a\left(s\right)\left(s+m_{0}\right)}=\frac{p_{num}\left(s\right)}{m\left(s\right)\left(s+m_{0}\right){\left(b_{0}+b_{0D}\right)}^{2}}$$

Hence, the estimation of $W_{M}\left(s\right)$ followed by the selection of $W_{P}\left(s\right)$ and testing inequality (38) for the Original model, Model 1, the Best-fit model, and the Relay model is presented below.

**Fig. 11:**
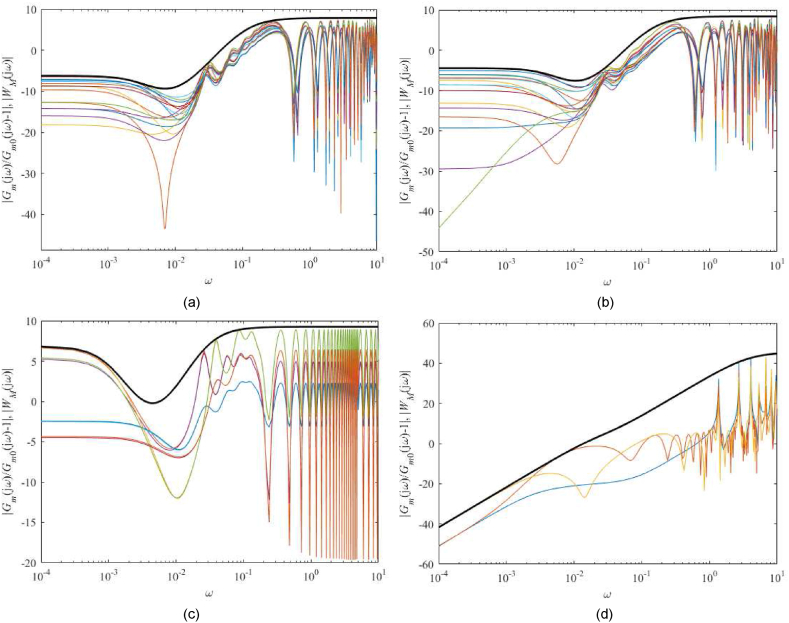
Bode plots of inequality test (33) for the Original model (a), Model 1 (b), the Best-fit model (c), and the Relay model (d). Perturbations (9), delay variations as in Table 3, and the ambient temperature range $\vartheta_{a}\in \left[18,28\right]$ °C are considered (color lines). The eventual upper bounds (40) are displayed (thick black line). (For interpretation of the references to color in this figure legend, the reader is referred to the Web version of this article.)

Fig. 11(a–d) displays the left-hand sides of (33) upper-bounded by $\left|W_{M}\left(j\omega \right)\right|$ that are constructed by virtue of the procedure described in [39] (see Section 4 therein). Perturbations (9), the corresponding delay variations in Table 3, and the ranging of the ambient temperature $\vartheta_{a}\in \left[18,28\right]$ °C are taken to calculate the set of all $G_{m}\left(s\right)$. For the Relay model, it is impossible to apply the above-given perturbations. Models “Relay2”, “Relay3”, and “Relay4” in Table 4 are taken as modelled perturbations in this case.

The eventual uncertainty weight functions $W_{M}\left(s\right)$ read

(40) $$\begin{array}{l} W_{M,\text{Original}}\left(s\right)=\frac{0.49{\left(121s+1\right)}^{2}}{\left(322s+1\right)\left(9s+1\right)},W_{M,\;\text{Model}1}\left(s\right)=\frac{0.6{\left(81s+1\right)}^{2}}{\left(215s+1\right)\left(7s+1\right)}\text{,}\\ W_{M,\text{Best}-\text{fit}}\left(s\right)=\frac{2.2{\left(217s+1\right)}^{2}}{\left(940s+1\right)\left(38s+1\right)},W_{M,\text{Relay}}\left(s\right)=\frac{82\left(30s+1\right)}{\left(51s+1\right)\left(0.26s+1\right)} \end{array}$$

Remark 2 Conditions (33), (36), and (38) are invariant to a change of the nominal model static gain. That is, whenever ${\tilde{G}}_{m,0}\left(s\right)=k_{0}G_{m,0}\left(s\right)$, then $k_{0}$ vanishes in these inequalities.

The following idea is used in the setting of $W_{P}\left(s\right)$ in (34). Let $s_{i}=-a$ be the dominant pole of $G_{m,0}\left(s\right)$. It is generally suggested to set it as the pole of $T_{0}\left(s\right)$. I.e., assume the approximation $T_{0}\left(s\right)\approx a/\left(s+a\right)$ that agrees with $S_{0}\left(s\right)\approx s/\left(s+a\right)$. That is, one can consider the upper bound in (34) as $W_{P}\left(s\right)\approx \left(s+a\right)/s$. However, numerical tests have shown that it is impossible to meet the condition; therefore, a weaker requirement

(41) $$W_{P}\left(s\right)\approx k_{W}\frac{s+a}{s},k_{W}\in \left(0,1\right)$$

is eventually taken, with a sufficient margin on (34). Hence, it is set $m_{0}=m_{1}=a$ for the nominal model controller (23)–(24) and $k_{W}$ is found so that ${\left\|S_{0}\left(j\omega \right)\right\|}_{\infty }=x{\left\|W_{P}\left(j\omega \right)\right\|}_{\infty }^{-1}$ where x≈0.6 is the selected margin (conservativeness). Table 5 summarizes the obtained results on (41).

**Table 5:** Dominant poles of nominal models and computed gains $k_{W}$ in (41) for (34) with 1DoF control system.

	Original	Model 1	Best-fit	Relay
$s_{i}=-a$ (x 10^−2^)	−2.682	−2.838	−2.841	−2.515
$k_{W}$	0.2	0.2	0.2	0.22

**Fig. 12:**
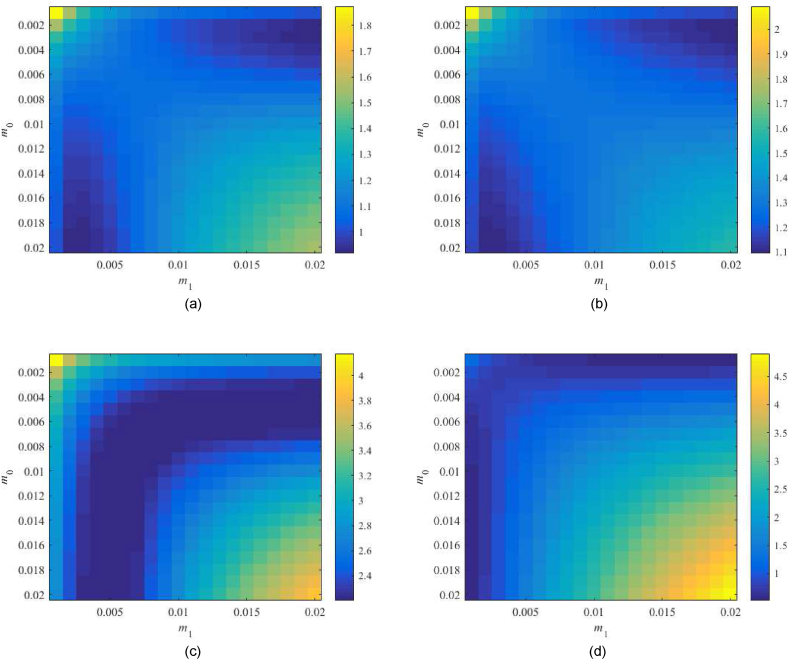
Value of $RP_{1\text{DoF}}$, see (38), for the parameter space $m_{0}\times m_{1}=\left[0.001,0.02\right]\times \left[0.001,0.02\right]$ with 1DoF; the Original model (a), Model 1 (b), the Best-fit model (c), and the Relay model (d).

Robust performance condition (38) is tested for the parameter space $m_{0}\times m_{1}=\left[0.001,0.02\right]\times \left[0.001,0.02\right]$ first. Values of $RP_{1\text{DoF}}$ are displayed in Fig. 12(a–d).

**Fig. 13:**
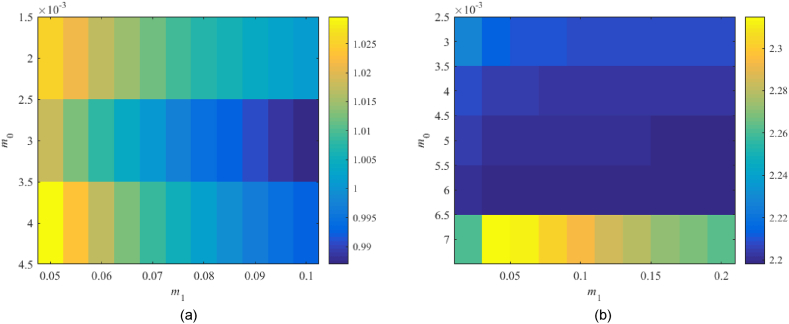
Value of $RP_{1\text{DoF}}$, see (38), for the extra parameter spaces $m_{0}\times m_{1}=\left[0.002,0.004\right]\times \left[0.05,0.1\right]$ (Model 1) – (a), and $m_{0}\times m_{1}=\left[0.003,0.007\right]\times \left[0.03,0.2\right]$ (Best-fit model) – (b) in 1DoF.

The Original model returns the minimum value of 0.930 for $m_{0}=0.003,m_{1}=0.018$ and the Relay model that of 0.615 at $m_{0}=0.002,m_{1}=0.011$, which represents satisfactory results. However, Model 1 returns the minimum value slightly above the performance border. The Best-fit model gives values much higher than 1. Therefore, two additional tests are performed in Fig. 13(a and b), namely, within the subspace $m_{0}\times m_{1}=\left[0.002,0.004\right]\times \left[0.05,0.1\right]$ for Model 1 and at $m_{0}\times m_{1}=\left[0.003,0.007\right]\times \left[0.03,0.2\right]$ for the Best-fit one.

In none of the two tests, a significant improvement has been obtained. Model 1 gives 0.987 for $m_{0}=0.003,m_{1}=0.01$ that is too close to the performance border yet satisfactory. The Best-fit model remains robustly unstable; hence, the robust performance cannot be met (the minimum is 2.198 at $m_{0}=0.006,m_{0}=0.2$). Besides, higher values of $m_{0},m_{1}$ mean faster control responses that usually imply excessive overshoots. The eventually selected controller parameters are summarized in Table 6.

**Table 6:** Selected parameters of controller (23)–(24) based on the robustness tests.

Parameter\model	Original	Model 1	Best-fit	Relay
$m_{0}$	0.003	0.003	0.006	0.002
$m_{1}$	0.018	0.1	0.2	0.011

### 5.2 Robustness design for TFC

The following lemmas hold for the TFC robust control design under the assumption that $G_{m,0}\left(s\right)$ and $G_{m}\left(s\right)$ have the equal number of poles *s*_*i*_ with $Res_{i}\geq 0$ [39,50].

Lemma 9 *The control system is robustly stable if and only if*

(42) $$<listaend>{\left\|RS_{\text{TFC}}\left(j\omega \right)\right\|}_{\infty }:={\left\|W_{M}\left(j\omega \right)T_{0}\left(j\omega \right)\left(1+\frac{C_{Q}\left(j\omega \right)}{C_{R}\left(j\omega \right)}\right)\right\|}_{\infty }<1$$

where the nominal complementary sensitivity function reads

(43) $$T_{0}\left(s\right)=\frac{C_{R}\left(s\right)G_{m,0}\left(s\right)}{1+\left[C_{Q}\left(s\right)+C_{R}\left(s\right)\right]G_{m,0}\left(s\right)}=B\left(s\right)R\left(s\right)=\frac{b\left(s\right)}{m\left(s\right)}\frac{m_{0}^{3}\left[\left(1-\lambda \right)v_{1}s^{2}+\left(v_{1}m_{1}+v_{0}\right)s+m_{1}v_{0}\right]}{{\left(b_{0}+b_{0D}\right)}^{2}\left(s+m_{0}\right)\left(s+m_{1}\right)}$$

with the nominal parameters in Table 4.

Lemma 10 The control system satisfies inequality (35) and robust stability condition (42) if and only if

(44) $$RP_{\text{TFC}}:={\left\|W_{P}\left(j\omega \right)\left(S_{0}\left(j\omega \right)+RS_{\text{TFC}}\left(j\omega \right)\right)\right\|}_{\infty }+{\left\|RS_{\text{TFC}}\left(j\omega \right)\right\|}_{\infty }<1$$

where $S_{0}$ is given by (45)

(45) $$S_{0}\left(s\right)=\frac{1+C_{Q}\left(s\right)G_{m,0}\left(s\right)}{1+\left[C_{Q}\left(s\right)+C_{R}\left(s\right)\right]G_{m,0}\left(s\right)}=A\left(s\right)P\left(s\right)+B\left(s\right)Q\left(s\right)=\frac{a\left(s\right)}{m\left(s\right)}\frac{p_{num}\left(s\right)}{{\left(b_{0}+b_{0D}\right)}^{2}a\left(s\right)\left(s+m_{0}\right)}+\frac{b\left(s\right)}{m\left(s\right)}\frac{m_{0}^{3}\lambda v_{1}s^{2}}{{\left(b_{0}+b_{0D}\right)}^{2}\left(s+m_{0}\right)\left(s+m_{1}\right)}=\frac{p_{num}\left(s\right)\left(s+m_{1}\right)+b\left(s\right)m_{0}^{3}\lambda v_{1}s^{2}}{{\left(b_{0}+b_{0D}\right)}^{2}m\left(s\right)\left(s+m_{0}\right)\left(s+m_{1}\right)}$$

Following the design step from the preceding subsection, uncertainty weight functions $W_{M}\left(s\right)$ are given by (40) as well since they do not depend on the used control system structure. Contrariwise, the choice of $W_{P}\left(s\right)$ can be different. When adopting its form (41), Table 7 is eventually obtained. It is worth noting that $m_{0}=m_{1}=a$ (see Table 6) has been set again, and the weighting parameter mid-value λ=0.5 in controller (30) is selected.

**Table 7:** *Computed gains*$k_{W}$*in*(41)*for* (34) *with TFC control system*.

	Original	Model 1	Best-fit	Relay
$k_{W}$	0.15	0.12	0.15	0.17

As controller (30)-(31) has three tunable parameters, three sets of the robust performance test are made, namely for λ=0.25, λ=0.5, and λ=0.75.

**Fig. 14:**
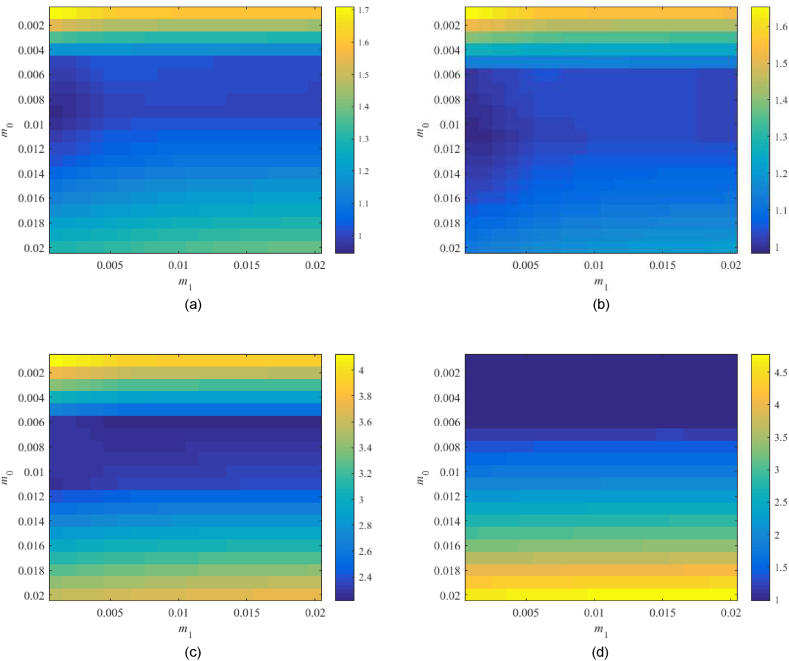
Value of $RP_{\text{TFC}}$, see (44), for the parameter space $m_{0}\times m_{1}=\left[0.001,0.02\right]\times \left[0.001,0.02\right]$ with λ=0.25 and TFC; the Original model (a), Model 1 (b), the Best-fit model (c), and the Relay model (d).

Consider λ=0.25 and $m_{0}\times m_{1}=$ [0.001,0.02]×[0.001,0.02] first. Fig. 14(a–d) displays values of the left-hand sides of robust performance condition (44).

For the Original model, the minimum value can be found for $m_{0}=0.009,m_{1}=0.001$ but a very low parameter value implies a slow control response. Note that point $m_{0}=0.009,m_{1}=0.002$ returns 0.956. Regarding Model 1, the minimum is 0.9811 at $m_{0}=0.011,m_{1}=0.001$; point $m_{0}=0.011,m_{1}=0.002$ gives 0.991 that is, however, close to the performance border. The robust performance of the Best-fit model cannot be satisfied again. Its minimum 2.213 is at $m_{0}=0.006,m_{1}=0.02$. The Relay model has a minimum 0.986 at $m_{0}=0.002,m_{1}=0.02$ and returns the value 0.999 for $m_{0}=0.0065\;m_{1}=0.002$, which attacks the border.

**Fig. 15:**
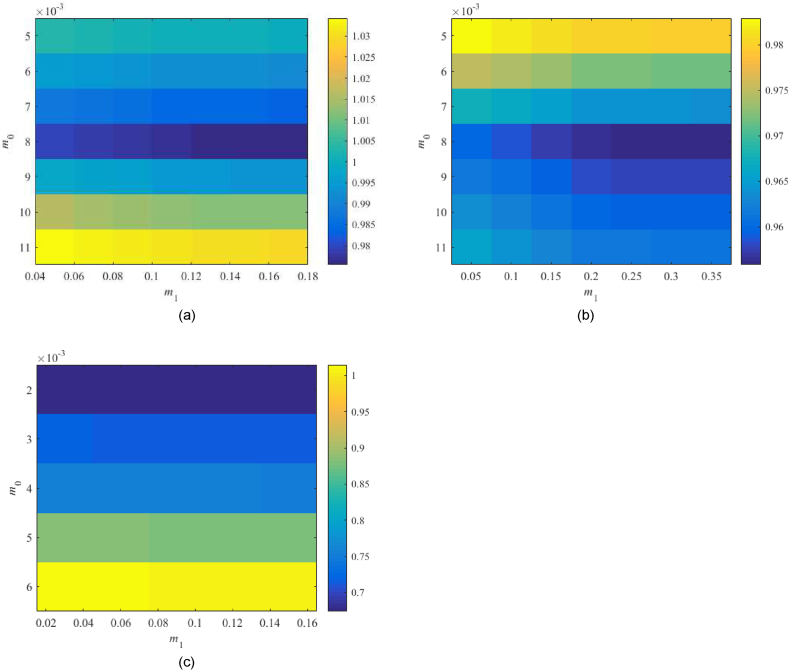
Value of $RP_{\text{TFC}}$, see (44), for the extra parameter spaces with λ=0.25 and TFC; the Original model (a), Model 1 (b), and the Relay model (c).

Based on the above-given data and Fig. 12(a–d), let us compute values for selected models in other subspaces. Namely, region $m_{0}\times m_{1}=\left[0.005,0.011\right]\times \left[0.05,0.17\right]$ is explored for the Original model, $m_{0}\times m_{1}=\left[0.005,0.011\right]\times \left[0.05,0.35\right]$ for Model 1, and $m_{0}\times m_{1}=\left[0.002,0.006\right]\times \left[0.03,0.15\right]$ for the Relay model. The corresponding results are provided in Fig. 15(a–c).

The found minima are the following: 0.976 at $m_{0}=0.008,m_{1}=0.17$ for the Original model, 0.956 at $m_{0}=0.008,m_{1}=0.3$ for Model 1, and 0.674 at $m_{0}=0.002,m_{1}=0.15$ for the Relay model. Simulations, however, have proven that high values of $m_{1}$ (with regard to the process dominant time constant) give aggressive control responses with high overshoots.

**Fig. 16:**
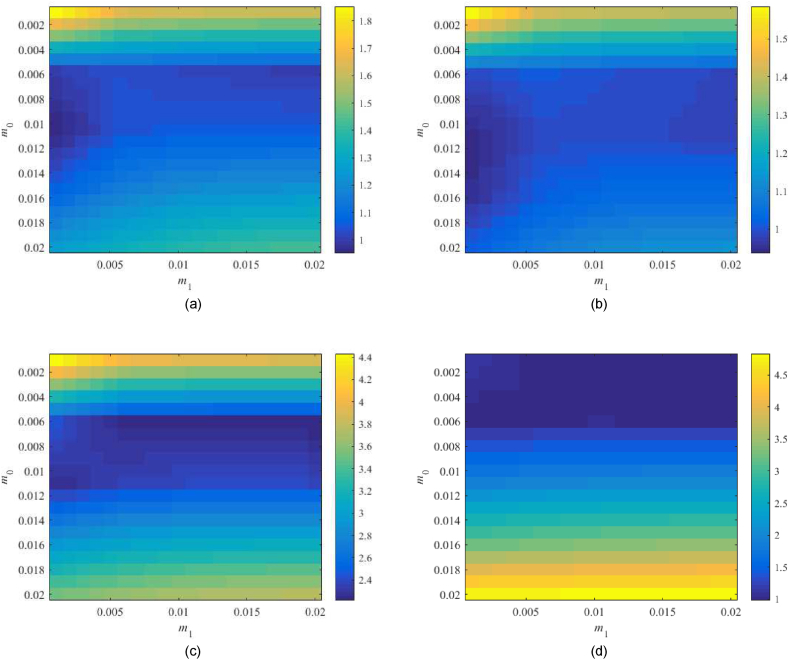
Value of $RP_{\text{TFC}}$, see (44), for the parameter space $m_{0}\times m_{1}=\left[0.001,0.02\right]\times \left[0.001,0.02\right]$ with λ=0.5 and TFC; the Original model (a), Model 1 (b), the Best-fit model (c), and the Relay model (d).

Now, assume λ=0.5 and $m_{0}\times m_{1}=$ [0.001,0.02]×[0.001,0.02]. Values of the left-hand sides of (44) are given in Fig. 16(a–d).

The observed minima and some other selected subspace points and values are as follows: The Original model returns 0.951 at $m_{0}=0.011,m_{1}=0.001$, and 0.969 at $m_{0}=0.011,m_{1}=0.002$; Model 1 gives 0.937 at $m_{0}=0.011,m_{1}=0.001$ and 0.948 at $m_{0}=0.011\text{,}$ $m_{1}=0.002$; the Best-fit model is far beyond the robust performance border with 2.217 at $m_{0}=0.006,m_{1}=0.02$; and the Relay model returns 0.990 at $m_{0}=0.006,m_{1}=0.002$.

It is worth noting that the robust performance problem with the Best-fit model cannot be solved by a less conservative selection of $W_{P}\left(s\right)$ as the problem is caused by the robust-stability term in (44).

**Fig. 17:**
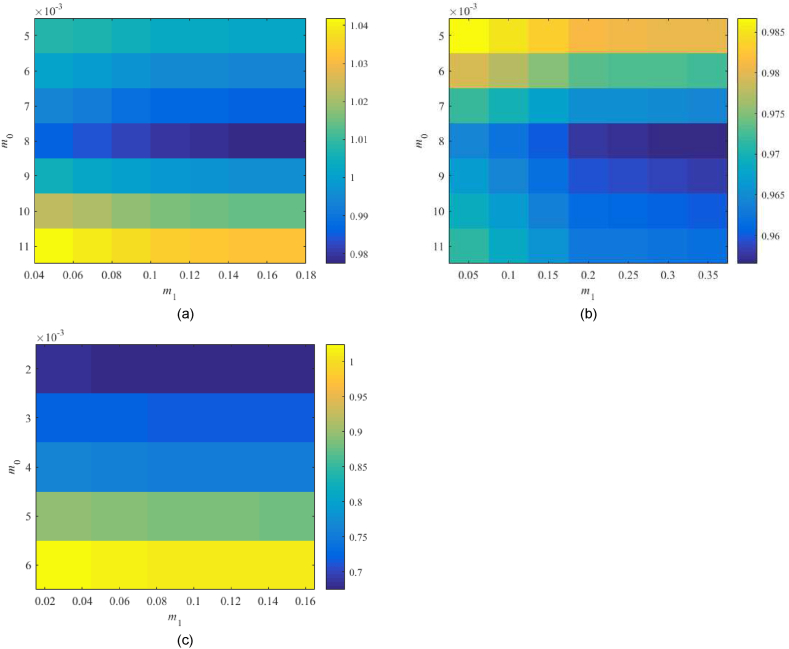
*Value of*$RP_{\text{TFC}}$, *see* (44), *for the extra parameter spaces with*λ=0.5*and TFC*; *the Original model* (*a*), *Model* 1 (*b*), *and the Relay model* (*c*).

Again, let us attempt to inspect also other parameter subspaces. Namely, region $m_{0}\times m_{1}=$ [0.005,0.011]×[0.05,0.17] is explored for the Original model, $m_{0}\times m_{1}=\left[0.005,0.011\right]\times \left[0.05,0.35\right]$ for Model 1, and $m_{0}\times m_{1}=\left[0.002,0.006\right]\times \left[0.03,0.15\right]$ for the Relay model. The corresponding data are provided in Fig. 17(a–c). The found minima are the following: 0.977 at $m_{0}=0.008,m_{1}=0.17$ for the Original model, 0.957 at $m_{0}=0.008,m_{1}=0.3$ for Model 1, and 0.675 at $m_{0}=0.002,m_{1}=0.15$ for the Relay model, which are data very similar to those of λ=0.25.

**Fig. 18:**
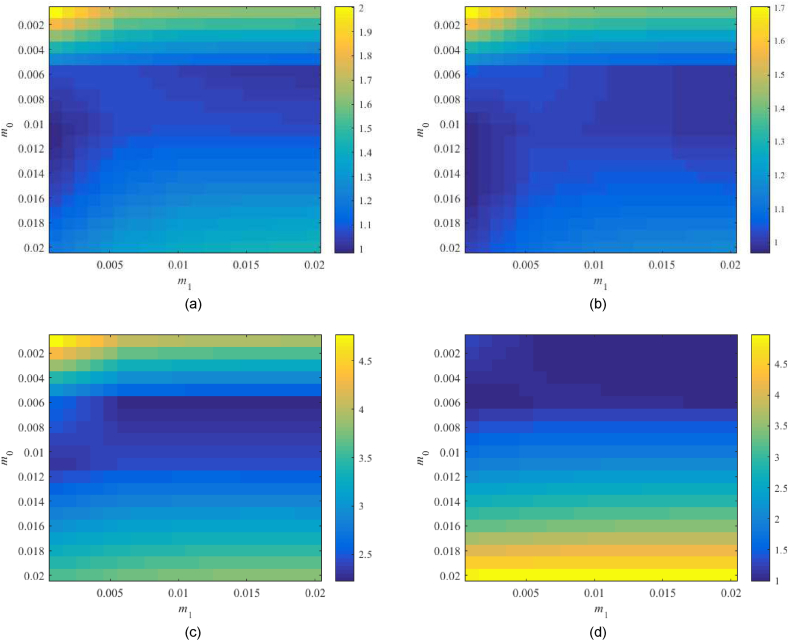
Value of $RP_{\text{TFC}}$, see (44), for the parameter space $m_{0}\times m_{1}=\left[0.001,0.02\right]\times \left[0.001,0.02\right]$ with λ=0.75 and TFC; the Original model (a), Model 1 (b), Best-fit model (c), and the Relay model (d).

Finally, let λ=0.75 be taken. Results of (44) for $m_{0}\times m_{1}=\left[0.001,0.02\right]\times \left[0.001,0.02\right]$ are given in Fig. 18(a–d).

The Original model returns the minimum 0.982 at $m_{0}=0.011,m_{1}=0.001$ but point $m_{0}=0.011,m_{1}=0.002$ has a value of 1.004 that does not satisfy (44). Model 1 gives 0.983 at $m_{0}=0.016,m_{1}=0.002$, while point $m_{0}=0.011,m_{1}=0.002$ returns a worse value of 0.984. The Best-fit model is far beyond the robust performance border again (with a minimum value of 2.221 at $m_{0}=0.006,m_{1}=0.02$). Finally, the Relay model returns 0.889 at $m_{0}=0.006,m_{1}=0.001$; however, the adjacent point $m_{0}=0.006,m_{1}=0.002$ has an unacceptable value of 1.004.

Some other parametric space regions have been computationally explored again, providing better performance values according to (44); however, due to a high control action and a high-speed control action are not suitable for the eventual control experiments. Note that particular robust performance measure values are similar to those for λ=0.25 and λ=0.5.

Based on the analysis above, the parameters of controller (30)-(31) provided to the reader in Table 8 have eventually been chosen for further laboratory control experiments.

**Table 8:** Selected parameters of controller (30)-(31) based on the robustness tests.

Model\parameter	λ	$m_{0}$	$m_{1}$
Original	0.3	0.01	0.002
	0.7	0.011	0.001
Model 1	0.3	0.011	0.002
	0.7	0.016	0.002
Best-fit	0.3	0.006	0.02
	0.7	0.006	0.02
Relay	0.3	0.005	0.002
	0.7	0.006	0.001

## 6 Laboratory control experiments

Experimental verification of robust controllers designed in Sections 4, 5 for the 1DoF and TFC control systems follows. The nominal case and several perturbations are considered. The received responses are then evaluated using some performance measures.

Denote controlled plant inputs and outputs by u(t)=ΔPHs(t), y(t)=ΔϑCOs(t), respectively, for simplicity. That is, the zero input-output values agree with the steady state (14). It i.a. means that the feasible range of u(t) is [−300,450] W. The reference signal is selected as follows: r(t)=0 (i.e., $P_{H}\left(t\right)=34.92$ °C) for time intervals [0,200), [4200,8200), and [20200,28000] s, r(t)=7 (i.e., $P_{H}\left(t\right)=41.92$ °C) for time intervals [201,4200) and [12200,16200) s, r(t) is linearly increasing from 0 to 7 within the interval [8200,12200) s and decreasing within [16200,20200) s. The constant load disturbance of d(t)=d=−50 W enters at t=24200 s, and it can represent power fluctuations of the electronic control circuit or a general ambiance effect.

### 6.1 Control responses for 1DoF

**Fig. 19:**
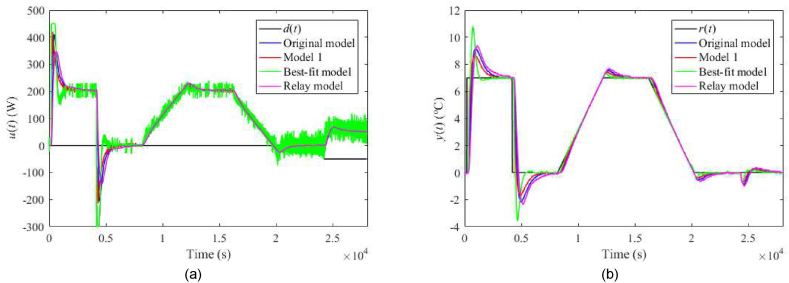
Control responses of u(t) (a) and y(t) (b) for the 1DoF nominal case in the neighbourhood of the operating point (14).

**Fig. 20:**
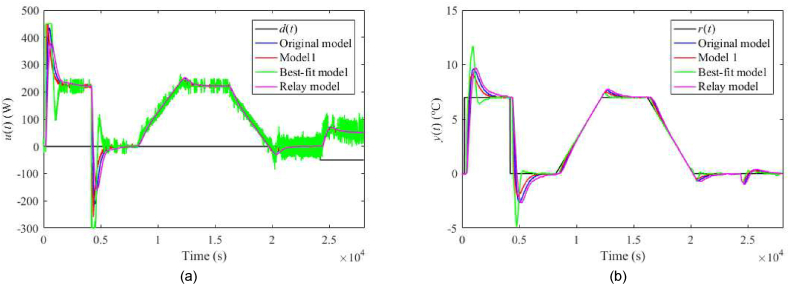
Control responses of u(t) (a) and y(t) (b) for the 1DoF perturbed case with $u_{P}\left(t\right)=4$ V.

**Fig. 21:**
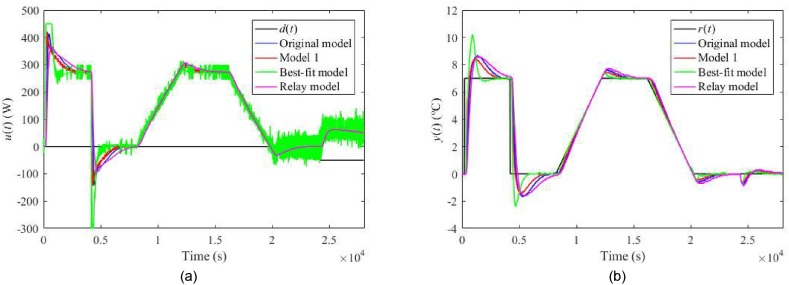
Control responses of u(t) (a) and y(t) (b) for the 1DoF perturbed case with $u_{C}\left(t\right)=4$ V.

**Fig. 22:**
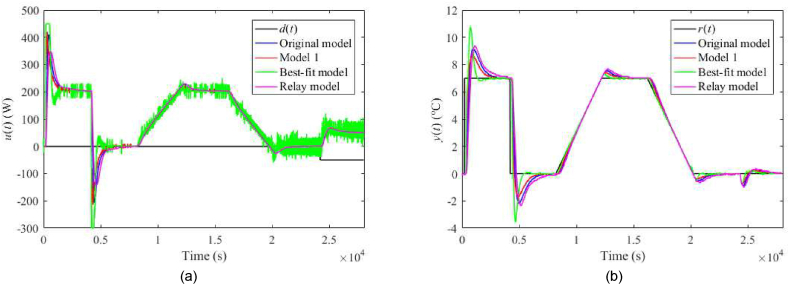
Control responses of u(t) (a) and y(t) (b) for the 1DoF perturbed case with $\vartheta_{a}\left(t\right)=21$ °C.

The nominal responses of u(t) and y(t) are displayed in Fig. 19(a and b). Now, consider a perturbation of $u_{P}\left(t\right)=4$ V (instead of the nominal value $u_{P}\left(t\right)=5$ V). Then, the corresponding control responses are provided in Fig. 20(a and b). Note that such a perturbation significantly impacts process delays. Let another process perturbation be $u_{C}\left(t\right)=4$ V (instead of the nominal value $u_{C}\left(t\right)=3$ V), see Fig. 21(a and b). Finally, let us test a decreased ambient temperature $\vartheta_{a}\left(t\right)=21$ °C (instead of the nominal value $\vartheta_{a}\left(t\right)=24$ °C) ensured by a room thermostat; see Fig. 22(a and b).

### 6.2 Control responses for TFC

**Fig. 23:**
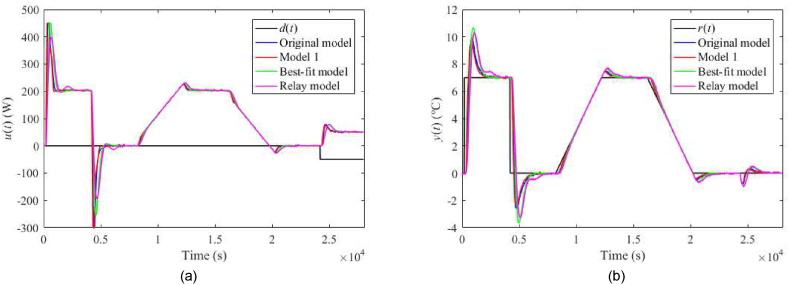
Control responses of u(t) (a) and y(t) (b) for the TFC nominal case with λ=0.3 in the neighbourhood of the operating point (14).

**Fig. 24:**
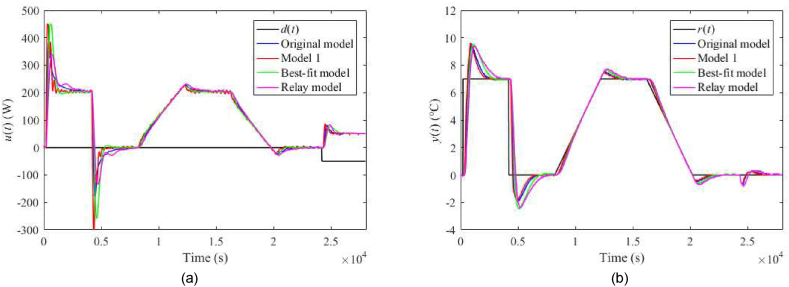
Control responses of u(t) (a) and y(t) (b) for the TFC nominal case with λ=0.7 in the neighbourhood of the operating point (14).

**Fig. 25:**
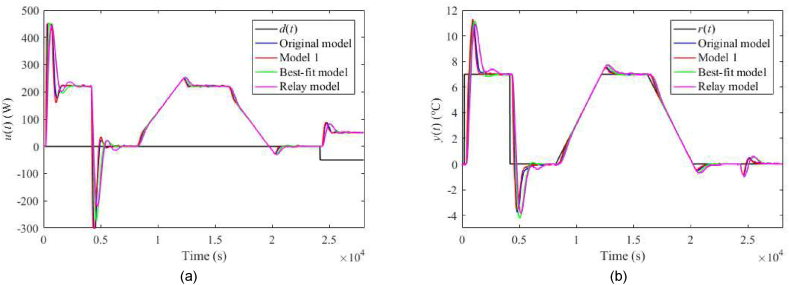
Control responses of u(t) (a) and y(t) (b) for the TFC perturbed case with $u_{P}\left(t\right)=4$ V and λ=0.3 in the neighbourhood of the operating point (14).

**Fig. 26:**
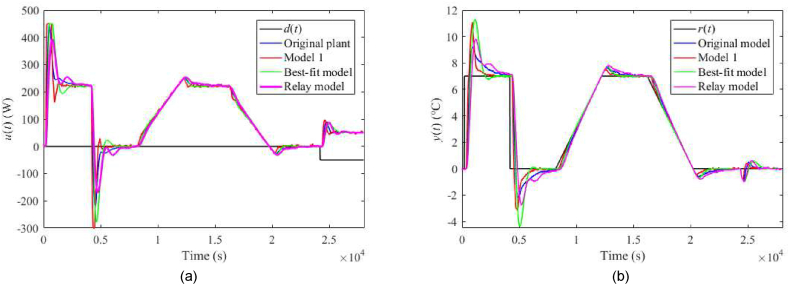
Control responses of u(t) (a) and y(t) (b) for the TFC perturbed case with $u_{P}\left(t\right)=4$ V and λ=0.7 in the neighbourhood of the operating point (14).

**Fig. 27:**
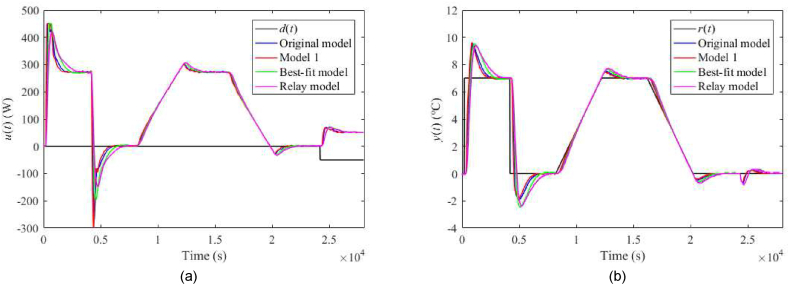
Control responses of u(t) (a) and y(t) (b) for the TFC perturbed case with $u_{C}\left(t\right)=4$ V and λ=0.3 in the neighbourhood of the operating point (14).

**Fig. 28:**
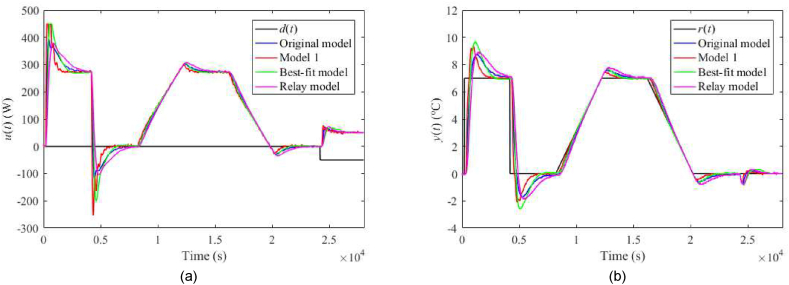
Control responses of u(t) (a) and y(t) (b) for the TFC perturbed case with $u_{C}\left(t\right)=4$ V and λ=0.7 in the neighbourhood of the operating point (14).

**Fig. 29:**
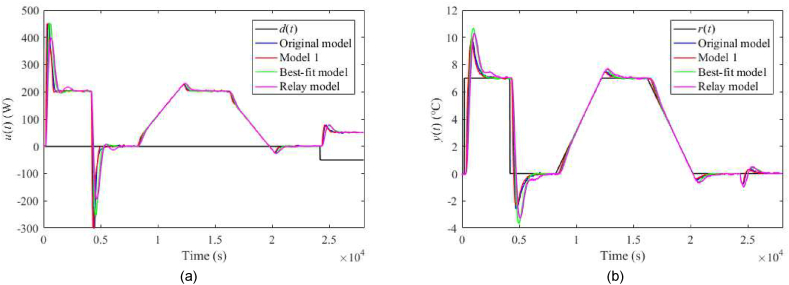
Control responses of u(t) (a) and y(t) (b) for the TFC perturbed case with $\vartheta_{a}\left(t\right)=21$ °C and λ=0.3 in the neighbourhood of the operating point (14).

**Fig. 30:**
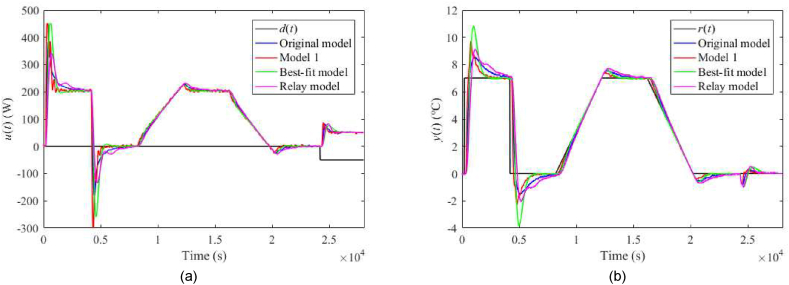
Control responses of u(t) (a) and y(t) (b) for the TFC perturbed case with $\vartheta_{a}\left(t\right)=21$ °C and λ=0.7 in the neighbourhood of the operating point (14).

The nominal responses of u(t) and y(t) for λ=0.3 and λ=0.7 are displayed in Fig. 23(a and b) and Fig. 24(a and b), respectively. Responses for the perturbed case $u_{P}\left(t\right)=4$ V with λ=0.3 and λ=0.7 are given in Fig. 25(a and b) and Fig. 26(a and b), respectively. Control performance under perturbation $u_{C}\left(t\right)=4$ with λ=0.3 and λ=0.7 are displayed in Fig. 27(a and b) and Fig. 28(a and b), respectively. Finally, control responses under the ambient temperature perturbation $\vartheta_{a}\left(t\right)=21$ °C for λ=0.3 and λ=0.7 are displayed in Fig. 29(a and b) and Fig. 30(a and b), respectively.

### 6.3 Experimental results evaluation

Several metrics are used for the evaluation of the control responses. Besides the IAE (12), output temperature overshoots/undershoots ($\Delta y_{\max}$) are measured, and three forms of the total variation (TV) are computed [55,56] to evaluate the monotonicity and deviation from it.

Controlled outputs are subject to an adjusted TV (denoted by $TV_{1}$ here in (46)) that expresses the difference between the total signal path and the minimum possible path required to change the signal from the initial state $y_{k_{0}}$ to the final state $y_{k_{1}}$:

(46) $$TV_{1}\left(y\right)=\sum_{k=k_{0}}^{k_{1}-1}\left|y_{k+1}-y_{k}\right|-\left|y_{k_{1}}-y_{k_{0}}\right|$$

It holds that the value of $TV_{1}\left(y\right)$ equals zero for monotonic outputs.

The basic TV (denoted by $TV_{0}$ here in (47))

(47) $$TV_{0}\left(u\right)=\sum_{k=k_{0}}^{k_{1}-1}\left|u_{k+1}-u_{k}\right|$$

is used to control action u(t) in those intervals, where the reference signal is linear-wise, as another one

(48) $$TV_{2}\left(u\right)=\sum_{k=k_{0}}^{k_{1}-1}\left|u_{k+1}-u_{k}\right|-\left|2u_{extr}-u_{k_{1}}-u_{k_{0}}\right|$$

is applicable for a step-wise r(t), where $u_{extr}$ is an extreme value lying $u_{extr}\notin \left(u_{k_{0}},u_{k_{1}}\right)$, i.e., outside the interval given by the initial and final values. $TV_{2}\left(u\right)$ in (48) expresses the deviation from an ideal single-pulse waveform (composed of two monotonic intervals) based on the Feldbaum theorem [55]. TVs for the control action mean the control effort, which, i.a., corresponds to the actuators’ lifetime.

In addition, the integral of $P_{H}\left(t\right)$ is evaluated

(49) $$EC\left(P_{H}\right)=\Delta t\sum_{k=k_{0}}^{k_{1}-1}{P_{H,}}_{k}$$

since (49) represents energy consumption, which is closely related to a very topical issue of sustainability and energy saving.

To evaluate the effects of step-wise and linear-wise reference changes, their increase and decrease, and the impact of load disturbance separately, let us calculate the performance measures for five disjoint time intervals:

(50) $$I_{1}\in \left[200,4200\right)s,I_{2}\in \left[4200,8200\right)s,I_{3}\in \left[8200,16200\right)s,I_{4}\in \left[16200,24200\right)s,I_{5}\in \left[24200,28000\right)s$$

Then, particular $k_{0}$ and $k_{1}$ correspond to the initial and final values, respectively, of intervals (50). Notice that $I_{5}$ serves only to the evaluation of the disturbance effect. Besides, the total IAE and EC for y(t) and $P_{H}\left(t\right)$, respectively, are calculated.

The results are summarized in Table F1 (1DoF), Table F2 (TFC, λ=0.3), and Table F3 (TFC, λ=0.7) that can be found in Appendix. Note that the values of $EC\left(P_{H}\right)$ are in kWh in the tables.

Let us select some distinct observations from the data. Regarding the 1DoF control system, relatively high values of $m_{0}$ or $m_{1}$ (see also Table 6) yield a chattering of u(t), which results in enormous values of $TV_{0}\left(u\right)$ and $TV_{2}\left(u\right)$, and also higher overshoots/undershoots. Model 1 and the Best-fit models give low IAEs in the nominal case, but the latter causes a high overshoot after the reference step change. The Best-fit model also provides the best output reaction to the load disturbance (IAE and $\Delta y_{\max}$). On the other side, this model is susceptible to perturbations (compared to the other models), which confirms the hypothesis that an almost exact nominal model might not be useful for robust control design and real-world control under uncertainties. The change of $u_{P}$ represents the most distinctive perturbation, mainly due to the change of delays (see Table 3). On the contrary, the responses are almost insensitive to an ambient temperature change. The data shows that Model 1 provides excellent responses under perturbations. Surprisingly, despite high IAEs given by low $m_{0},m_{1}$, the Relay model proves to be sufficient enough for control objectives. An unpleasant effect of perturbations and “fast” controller settings is the existence of the wind-up effect on u(t) that reaches its physical limits. Notice that the Best-fit model causes the wind-up even in the nominal case (Fig. 19(a,b)). There exist several principles to tackle this problem, e.g., a functional approach for time-delay systems [29]. This problem represents one of the tasks of our future research.

Regarding the TFC control system with λ=0.3, IAEs for the Original model give better values compared to 1DoF for step-wise reference changes; however, it does not hold for other models. Contrariwise, Model 1 and the Relay model yield lower IAEs when linear-wise reference tracking. A significant undesirable effect of TFC is an increase in overshoots/undershoots after a step change of r(t). Hence, the suggestion here is to use a filtered reference signal without abrupt changes in practice. In the cases of the Original model and Model 1, a better response to the load disturbance can be observed. An interesting observation can be made on the energy consumption: Its value seems almost invariant to the used model and control system and depends solely on a particular perturbation.

The setting λ=0.7 brings about higher IAEs but lower overshoots after the reference change compared to λ=0.3. Contrariwise, IAEs given be a reaction to the disturbance are better. Overall, the TFC control system cannot be indicated as better compared to the 1DoF structure. However, the possibility of tuning λ value brings more options when finding a trade-off between reference tracking and disturbance rejection.

## 7 Link to the Smith predictor

Finally, let us concisely compare to the well-establish Smith dead-time compensator [51]. As the process model structure (16)–(17) includes the input-output delay, such a comparison can be made. We also point out a significant drawback of this approach.

**Fig. 31:**
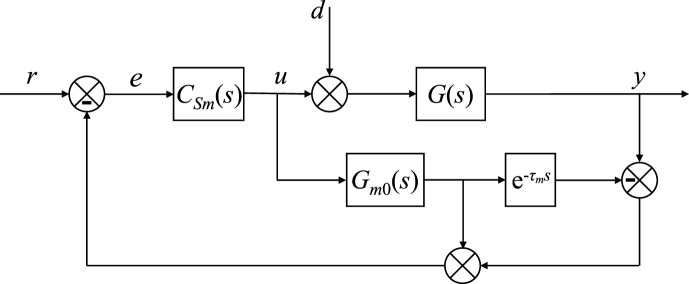
Smith predictor.

The Smith predictor structure is depicted in Fig. 31.

In the scheme, $G_{m0}\left(s\right)$ means a process model transfer function without the input-output delay. This modelled delay (i.e., its estimation) is represented by block $e^{-\tau_{m}s}$. The Smith-predictor controller has transfer function $C_{Sm}\left(s\right)$. Two research questions arise:

1) What is the relation between $C_{Sm}\left(s\right)$ and $C_{R}\left(s\right)$, $C_{Q}\left(s\right)$?
2) Does $C_{Sm}\left(s\right)$ work well?

For the 1DoF control system, the following relation can be derived based on the matching of reference-to-output transfer functions (i.e., T(s))

(51) $$C_{Sm,1\text{DoF}}\left(s\right)=\frac{C_{R}\left(s\right)}{1-C_{R}\left(s\right)G_{m0}\left(s\right)\left(1-e^{-\tau s}\right)}$$

See Appendix F for a sketch of the proof. Note that controller $C_{R}\left(s\right)$ is given by (23), (24)

For the TFC control system, the analogous matching yields

(52) $$C_{Sm,\text{TFC}}\left(s\right)=\frac{C_{R}\left(s\right)}{1-C_{R}\left(s\right)G_{m0}\left(s\right)\left(1-e^{-\tau s}\right)+G\left(s\right)C_{Q}\left(s\right)}$$

The reader is referred to Appendix F again. Transfer functions of controllers $C_{R}\left(s\right)$, $C_{Q}\left(s\right)$ for the heating-cooling process in question can be found in (30), (31).

It is worth noting that whereas (51) is independent of the actual process dynamics, controller (52) also depends on G(s). It implies that the agreement of TFC and the Smith predictor depends on an accurate estimate of the process dynamics.

Settings (51) and (52), however, bring about a significant drawback of this design:

Theorem 1 *The use of the Smith predictor with controllers* (51) *and* (52) *for process models* (23)*-*(24) *and* (30)*-*(31), *respectively*, *cannot guarantee an asymptotic linear-wise load disturbance rejection*.

*Proof of*Theorem 1*can also be found in Appendix G*. *It is worth noting that the designed* 1*DoF control system rejects the linear-wise disturbance*; *however*, *TFC does not*, *as indicated in the appendix as well*.

## 8 Conclusions

A detailed modelling, identification, and control study has been presented in this paper. A laboratory looped heating-cooling system with significant input-output and internal delays has been considered as the process in question. A thorough revision and reformulation of the model parameters identification procedure have been designed, resulting in a novel process model reflecting process and measurement uncertainties and perturbations. Besides, an accurate nominal model has been assembled as well for comparison. The control design has included a controller structure derivation for two different control systems, namely the 1DoF and the TFC structures, and the application of robust stability and robust performance conditions in detail. As a result, suitable controller parameter settings have been obtained. Laboratory experiments have proven the applicability of the controllers proposed based on the robust model in the nominal case and for several process perturbations. Moreover, comparisons to some other alternative models have been made. Namely, a controller designed based on a model obtained from a recent relay-feedback experiment has also proven to be capable of controlling the process under perturbations and a load disturbance. Contrariwise, a controller that best matches the nominal process data could not meet the robust performance conditions. A link to the well-established Smith dead-time compensator has concisely been introduced, and issues with process model estimation and disturbance attenuation have been highlighted.

The main findings of this research can be summarized as follows:

1) The static-parameter approximation error of the proposed model (Model 1) is less than the Original model.
2) Operating ranges of process delays have been estimated based on the dynamic-parameter identification for all considered models.
3) The Relay model has evinced the worst dynamic responses when matching the measured data.
4) The Best-fit model could not meet the robust stability and robust performance conditions for both control system structures. Model 1 has been very close to the borders for the 1DoF control system.
5) The Original and Relay models have satisfied the robust performance condition for slow control response settings in the TFC structure.
6) The Original model has given better step-wise reference with the TFC structure than the 1DoF scheme.
7) The Best-fit model matching the nominal step responses most accurate has provided poor robust control performance and could not be used for real-world control under uncertainties.
8) Relay model has proven to be sufficient enough for control purposes.
9) Model 1 has resulted in a lower IAE when linear-wise reference tracking with the TFC control system.
10) TFC has improved a load disturbance response but increased overshoots/undershoots after a reference step change compared to 1DoF.
11) The change of the pump input voltage has represented the most distinctive perturbation, mainly due to the change of delays.
12) Overall energy consumption has remained almost invariant to the used model and control system and depended only on perturbations.
13) The value of λ can serve as a tuning knob to reach a trade-off between reference tracking and disturbance rejection.
14) The analogous Smith predictor could not satisfy asymptotic linear-wise load disturbance rejection.

Possible future research can concern overshoot reduction (e.g., based on a model predictive control framework), anti-wind-up protection, a multivariable control design [57], or the use of a multiloop control system (with the auxiliary controlled variable), or the implementation of advanced parameter optimization techniques when identification [58]. In any case, we are motivated by a constant effort to create advanced modelling and control techniques to reduce energy dependence while maintaining sufficient user comfort.

## Author contribution statement

Libor Pekař: Conceived and designed the experiments; Performed the experiments; Analyzed and interpreted the data; Contributed reagents, materials, analysis tools or data; Wrote the paper.

Radek Matušů: Conceived and designed the experiments; Analyzed and interpreted the data; Wrote the paper.

Petr Dostálek: Performed the experiments; Wrote the paper.

Mengjie Song: Analyzed and interpreted the data; Contributed reagents, materials, analysis tools or data; Wrote the paper.

## Data availability statement

Data will be made available on request.

## Declaration of competing interest

The authors declare the following financial interests/personal relationships which may be considered as potential competing interests: Libor Pekar reports financial support was provided by Tomas Bata University in Zlin. Libor Pekar reports financial support was provided by College of Polytechnics Jihlava. Libor Pekar reports a relationship with Tomas Bata University in Zlin that includes: employment.

## Figures and Tables

**Table A.1:** Measured steady-state temperature values for various constant inputs ($\vartheta_{a}=24$ °C).

uPs (V)	uCs(V)	PHs(W)	ϑHOs(°C)	ϑCIs(°C)	ϑCOs(°C)
4	2	300	44.95	44.80	36.20
4	2	450	57.33	57.20	45.95
4	3	225	39.95	39.70	33.20
4	3	300	43.05	42.80	34.30
4	3	375	48.10	47.90	37.10
4	3	400	51.20	50.80	37.70
4	4	300	41.25	41.10	32.40
4	4	375	47.40	47.20	36.40
5	1	225	41.90	41.60	35.40
5	1	300	47.46	47.19	39.37
5	1	375	52.97	52.70	42.77
5	2	225	38.98	38.80	32.44
5	2	300	44.75	44.40	36.55
5	2	450	57.10	56.80	46.30
5	3	225	38.65	38.60	32.10
5	3	300	43.22	43.00	34.92
5	3	375	45.50	45.50	34.80
5	3	400	52.00	51.70	40.10
5	3	450	55.20	54.90	44.20
5	3	525	61.50	61.00	48.70
5	3	600	67.40	66.70	54.40
5	4	225	37.35	37.25	30.77
5	4	300	41.74	41.50	33.35
5	4	400	45.60	45.40	33.10
5	5	300	40.80	40.60	32.40
5	5	450	50.40	50.10	39.00
5	6	225	35.10	35.05	28.10
5	6	300	40.56	40.37	32.11
6	2	300	45.00	44.75	37.00
6	3	225	38.55	38.53	32.80
6	3	300	43.30	42.80	35.20
6	3	375	48.46	48.09	38.64
6	3	400	49.40	49.10	39.40
6	4	300	41.30	41.30	33.10

**Table F.1:** Experimental control responses evaluation for 1DoF.

Model Process	Original Nominal	Original Perturb. *u*_P_	Original Perturb. *u*_C_	Original Perturb. *ϑ*_*a*_	Model 1 Nominal	Model 1 Perturb. *u*_P_	Model 1 Perturb. *u*_C_	Model 1 Perturb. *ϑ*_*a*_
$IAE_{I}\left(y\right)$	4344	4893	4713	4345	3194	3666	3354	3193
$IAE_{\text{II}}\left(y\right)$	4314	4836	4694	4315	3150	3603	3487	3150
$IAE_{\text{III}}\left(y\right)$	1194	1301	1549	1195	875	941	1101	874
$IAE_{\text{IV}}\left(y\right)$	1137	1229	1538	1138	809	858	1060	803
$IAE_{I-\text{IV}}\left(y\right)$	10990	12259	12494	10991	8028	9068	9183	8021
$IAE_{V}\left(y\right)$	786.65	843.08	717.45	786.56	571.73	612.32	560.44	540.04
$TV_{1,I}\left(y\right)$	6.34	8.32	5.15	6.14	7.13	7.92	6.93	6.93
$TV_{1,\text{II}}\left(y\right)$	8.12	8.12	5.94	8.12	7.52	8.12	5.15	7.13
$TV_{1,\text{III}}\left(y\right)$	9.31	9.90	10.89	9.31	11.48	9.70	9.50	12.47
$TV_{1,\text{IV}}\left(y\right)$	8.71	6.93	7.33	8.71	18.22	12.47	14.65	16.43
$TV_{2,I}\left(u\right)$	577	473	554	577	1603	1610	1623	1591
$TV_{2,\text{II}}\left(u\right)$	553	429	292	553	2200	2348	1938	2128
$TV_{0,\text{III}}\left(u\right)$	292	317	366	293	3185	2871	2941	3278
$TV_{0,\text{IV}}\left(u\right)$	283	300	353	283	8696	8235	1025	8606
$TV_{0,V}\left(u\right)$	101	107	90	101	2024	1809	1902	1617
$EC_{I-\text{IV}}\left(P_{H}\right)$	2.679	2.740	2.912	2.699	2.680	2.739	2.912	2.680
$EC_{V}\left(P_{H}\right)$	0.370	0.371	0.372	0.371	0.371	0.371	0.371	0.370
$\Delta y_{\max,I}$	2.11	2.60	1.71	2.13	1.71	2.11	1.42	1.71
$\Delta y_{\max,\text{II}}$	2.18	2.67	1.68	2.21	1.68	2.08	1.49	1.68
$\Delta y_{\max,\text{III}}$	0.62	0.62	0.62	0.63	0.43	0.52	0.43	0.44
$\Delta y_{\max,\text{IV}}$	0.59	0.59	0.59	0.59	0.40	0.50	0.50	0.39
$\Delta y_{\max,V}$	0.89	0.99	0.79	0.90	0.79	0.79	0.69	0.69

**Model** **Process**	**Best-fit** **Nominal**	**Best-fit** **Perturb.*****u***_**P**_	**Best-fit** **Perturb.*****u***_**C**_	**Best-fit** **Perturb.*****ϑ***_***a***_	**Relay** **Nominal**	**Relay** **Perturb.*****u***_**P**_	**Relay** **Perturb.*****u***_**C**_	**Relay** **Perturb.*****ϑ***_***a***_

$IAE_{I}\left(y\right)$	3053	4077	3378	3051	5170	5731	5659	5170
$IAE_{\text{II}}\left(y\right)$	2711	3441	2718	2692	5159	5725	5694	5159
$IAE_{\text{III}}\left(y\right)$	524	561	613	497	1709	1860	2321	1710
$IAE_{\text{IV}}\left(y\right)$	525	599	603	495	1689	1842	2301	1690
$IAE_{I-\text{IV}}\left(y\right)$	6813	8678	7311	6735	13727	15158	15975	13728
$IAE_{V}\left(y\right)$	419.76	545.59	415.70	426.49	976.34	1024.25	909.61	975.74
$TV_{1,I}\left(y\right)$	13.66	15.05	11.68	13.07	7.72	8.12	6.93	7.72
$TV_{1,\text{II}}\left(y\right)$	10.30	15.25	7.52	9.11	7.92	7.52	6.14	7.94
$TV_{1,\text{III}}\left(y\right)$	10.30	10.49	11.48	12.67	7.13	6.93	8.02	7.14
$TV_{1,\text{IV}}\left(y\right)$	22.57	10.30	15.25	15.84	9.90	9.50	14.26	9.91
$TV_{2,I}\left(u\right)$	5883	5366	4326	5605	126	118	59	126
$TV_{2,\text{II}}\left(u\right)$	8741	12247	8801	5276	289	318	184	290
$TV_{0,\text{III}}\left(u\right)$	10893	10484	11910	11425	263	288	345	263
$TV_{0,\text{IV}}\left(u\right)$	88940	88470	118682	87716	379	342	452	381
$TV_{0,V}\left(u\right)$	31033	27736	50217	29957	90	93	123	91
$EC_{I-\text{IV}}\left(P_{H}\right)$	2.677	2.734	2.904	2.676	2.680	2.741	2.912	2.681
$EC_{V}\left(P_{H}\right)$	0.361	0.361	0.356	0.360	0.370	0.370	0.371	0.370
$\Delta y_{\max,I}$	3.79	4.68	3.20	3.80	2.41	2.70	1.61	2.42
$\Delta y_{\max,\text{II}}$	3.56	4.85	2.38	3.58	2.38	2.67	1.58	2.38
$\Delta y_{\max,\text{III}}$	0.33	0.43	0.33	0.34	0.72	0.72	0.72	0.72
$\Delta y_{\max,\text{IV}}$	0.59	0.69	0.59	0.60	0.69	0.70	0.69	0.69
$\Delta y_{\max,V}$	0.69	0.79	0.69	0.79	0.99	1.01	0.90	1.00

**Table F.2:** Experimental control responses evaluation for TFC with λ=0.3.

Model Process	Original Nominal	Original Perturb. *u*_P_	Original Perturb. *u*_C_	Original Perturb. *ϑ*_*a*_	Model 1 Nominal	Model 1 Perturb. *u*_P_	Model 1 Perturb. *u*_C_	Model 1 Perturb. *ϑ*_*a*_
$IAE_{I}\left(y\right)$	3802	4338	4108	3803	3391	3920	3685	3391
$IAE_{\text{II}}\left(y\right)$	3731	4238	3910	3729	3292	3825	3448	3293
$IAE_{\text{III}}\left(y\right)$	855	899	1033	855	753	783	886	753
$IAE_{\text{IV}}\left(y\right)$	786	825	947	785	678	700	802	677
$IAE_{I-\text{IV}}\left(y\right)$	9173	10300	9998	9172	8113	9228	8821	8113
$IAE_{V}\left(y\right)$	506.48	565.42	457.81	506.79	424.12	488.07	389.27	424.01
$TV_{1,I}\left(y\right)$	10.30	10.49	9.70	10.29	9.11	11.29	8.12	9.10
$TV_{1,\text{II}}\left(y\right)$	8.71	10.30	7.13	8.91	7.92	10.49	7.33	7.92
$TV_{1,\text{III}}\left(y\right)$	7.72	8.51	9.01	7.73	7.92	10.00	11.48	7.72
$TV_{1,\text{IV}}\left(y\right)$	6.73	8.32	7.72	6.53	7.33	7.72	6.14	7.34
$TV_{2,I}\left(u\right)$	518	563	402	517	545	551	428	546
$TV_{2,\text{II}}\left(u\right)$	661	697	732	662	718	768	722	718
$TV_{0,\text{III}}\left(u\right)$	345	368	428	345	524	577	644	523
$TV_{0,\text{IV}}\left(u\right)$	321	346	391	323	484	506	534	484
$TV_{0,V}\left(u\right)$	141	158	120	141	176	195	156	176
$EC_{I-\text{IV}}\left(P_{H}\right)$	2.680	2.741	2.913	2.680	2.679	2.740	2.911	2.679
$EC_{V}\left(P_{H}\right)$	0.371	0.371	0.372	0.371	0.370	0.370	0.371	0.370
$\Delta y_{I}$	2.90	4.09	2.50	2.91	3.00	4.29	2.60	2.99
$\Delta y_{\text{II}}$	2.57	3.76	1.88	2.57	2.37	3.56	1.98	2.38
$\Delta y_{\text{III}}$	0.52	0.52	0.52	0.52	0.43	0.52	0.43	0.42
$\Delta y_{\text{IV}}$	0.50	0.50	0.50	0.49	0.40	0.50	0.40	0.41
$\Delta y_{V}$	0.79	0.89	0.70	0.79	0.79	0.79	0.69	0.80

**Model** **Process**	**Best-fit** **Nominal**	**Best-fit** **Perturb.*****u***_**P**_	**Best-fit** **Perturb.*****u***_**C**_	**Best-fit** **Perturb.*****ϑ***_***a***_	**Relay** **Nominal**	**Relay** **Perturb.*****u***_**P**_	**Relay** **Perturb.*****u***_**C**_	**Relay** **Perturb.*****ϑ***_***a***_

$IAE_{I}\left(y\right)$	4934	5689	5314	4934	5298	5858	5707	5298
$IAE_{\text{II}}\left(y\right)$	4854	5449	5264	4854	5301	5820	5683	5300
$IAE_{\text{III}}\left(y\right)$	1109	1220	1433	1110	1477	1573	1807	1478
$IAE_{\text{IV}}\left(y\right)$	1029	1107	1388	1031	1439	1507	1777	1440
$IAE_{I-\text{IV}}\left(y\right)$	11925	13465	13399	11929	13515	14758	14974	13516
$IAE_{V}\left(y\right)$	830.31	879.42	745.97	838.83	847.44	911.59	760.52	846.25
$TV_{1,I}\left(y\right)$	10.99	12.28	7.33	10.99	9.11	12.08	7.72	8.91
$TV_{1,\text{II}}\left(y\right)$	10.49	10.69	9.50	10.50	11.19	11.88	7.52	10.99
$TV_{1,\text{III}}\left(y\right)$	10.69	10.68	10.50	11.09	8.61	8.32	8.71	8.61
$TV_{1,\text{IV}}\left(y\right)$	6.34	6.14	8.51	6.53	10.10	7.13	5.74	10.10
$TV_{2,I}\left(u\right)$	605	721	614	605	211	227	104	212
$TV_{2,\text{II}}\left(u\right)$	412	346	402	411	437	520	297	437
$TV_{0,\text{III}}\left(u\right)$	278	305	352	278	273	300	350	273
$TV_{0,\text{IV}}\left(u\right)$	266	289	349	265	267	292	341	268
$TV_{0,V}\left(u\right)$	110	117	90	111	117	133	96	117
$EC_{I-\text{IV}}\left(P_{H}\right)$	2.679	2.740	2.913	2.679	2.680	2.740	2.912	2.680
$EC_{V}\left(P_{H}\right)$	0.371	0.371	0.371	0.371	0.370	0.371	0.371	0.370
$\Delta y_{\max,I}$	3.69	4.19	2.50	3.69	3.30	3.89	2.41	3.30
$\Delta y_{\max,\text{II}}$	3.66	4.26	2.48	3.66	3.27	3.86	2.38	3.27
$\Delta y_{\max,\text{III}}$	0.62	0.72	0.72	0.62	0.72	0.72	0.72	0.72
$\Delta y_{\max,\text{IV}}$	0.59	0.69	0.69	0.60	0.69	0.69	0.69	0.69
$\Delta y_{\max,V}$	1.00	1.00	0.89	0.99	0.99	0.99	0.79	0.99

**Table F.3:** Experimental control responses evaluation for TFC with λ=0.7.

Model Process	Original Nominal	Original Perturb. *u*_P_	Original Perturb. *u*_C_	Original Perturb. *ϑ*_*a*_	Model 1 Nominal	Model 1 Perturb. *u*_P_	Model 1 Perturb. *u*_C_	Model 1 Perturb. *ϑ*_*a*_
$IAE_{I}\left(y\right)$	4333	4828	4694	4333	3272	3789	3545	3272
$IAE_{\text{II}}\left(y\right)$	4325	4800	4692	4323	3200	3724	3380	3200
$IAE_{\text{III}}\left(y\right)$	1660	1689	1785	1662	824	836	887	814
$IAE_{\text{IV}}\left(y\right)$	1601	1637	1737	1600	742	780	814	740
$IAE_{I-\text{IV}}\left(y\right)$	11919	12954	12907	11917	8037	9129	8626	8026
$IAE_{V}\left(y\right)$	466.88	530.64	431.15	463.22	292.94	375.31	280.76	291.26
$TV_{1,I}\left(y\right)$	6.53	8.32	5.54	6.53	9.70	11.68	8.91	9.70
$TV_{1,\text{II}}\left(y\right)$	6.73	7.92	6.34	6.53	9.50	10.69	7.72	9.31
$TV_{1,\text{III}}\left(y\right)$	8.51	9.70	8.71	8.71	8.71	10.69	7.33	8.91
$TV_{1,\text{IV}}\left(y\right)$	9.31	11.68	9.70	9.31	7.33	8.32	7.72	8.12
$TV_{2,I}\left(u\right)$	587	506	519	590	1006	788	825	1006
$TV_{2,\text{II}}\left(u\right)$	422	453	305	420	1168	1112	1134	1166
$TV_{0,\text{III}}\left(u\right)$	375	410	444	378	1187	1268	1175	1180
$TV_{0,\text{IV}}\left(u\right)$	384	406	446	384	1045	1149	1133	1067
$TV_{0,V}\left(u\right)$	157	181	133	159	361	428	303	347
$EC_{I-\text{IV}}\left(P_{H}\right)$	2.680	2.741	2.912	2.680	2.679	2.741	2.912	2.680
$EC_{V}\left(P_{H}\right)$	0.370	0.371	0.371	0.370	0.371	0.370	0.370	0.370
$\Delta y_{\max,I}$	1.51	2.21	1.71	1.52	2.70	4.09	2.31	2.70
$\Delta y_{\max,\text{II}}$	1.49	2.18	1.78	1.49	2.28	3.17	2.08	2.28
$\Delta y_{\max,\text{III}}$	0.52	0.62	0.62	0.52	0.43	0.52	0.43	0.43
$\Delta y_{\max,\text{IV}}$	0.59	0.60	0.59	0.59	0.40	0.50	0.50	0.40
$\Delta y_{\max,V}$	0.79	0.89	0.69	0.79	0.79	0.79	0.69	0.79

**Model** **Process**	**Best-fit** **Nominal**	**Best-fit** **Perturb.*****u***_**P**_	**Best-fit** **Perturb.*****u***_**C**_	**Best-fit** **Perturb.*****ϑ***_***a***_	**Relay** **Nominal**	**Relay** **Perturb.*****u***_**P**_	**Relay** **Perturb.*****u***_**C**_	**Relay** **Perturb.*****ϑ***_***a***_

$IAE_{I}\left(y\right)$	5153	5934	5538	5152	5753	6259	6240	5753
$IAE_{\text{II}}\left(y\right)$	5076	5685	5483	5073	5744	6260	6231	5744
$IAE_{\text{III}}\left(y\right)$	1156	1269	1489	1155	2482	2546	2719	2482
$IAE_{\text{IV}}\left(y\right)$	1065	1161	1443	1065	2377	2434	2627	2376
$IAE_{I-\text{IV}}\left(y\right)$	12450	14409	13953	12446	16355	17498	17816	16354
$IAE_{V}\left(y\right)$	832.89	897.44	747.65	833.18	728.15	774.48	651.42	728.24
$TV_{1,I}\left(y\right)$	12.67	14.45	8.42	12.97	6.34	9.70	6.53	6.34
$TV_{1,\text{II}}\left(y\right)$	11.48	11.09	9.31	11.68	8.32	8.71	7.33	8.32
$TV_{1,\text{III}}\left(y\right)$	11.39	9.50	12.67	11.39	8.51	9.50	9.70	8.52
$TV_{1,\text{IV}}\left(y\right)$	6.53	5.94	8.32	6.73	7.92	8.71	7.33	7.92
$TV_{2,I}\left(u\right)$	618	726	621	617	144	185	58	144
$TV_{2,\text{II}}\left(u\right)$	408	358	413	411	294	403	211	294
$TV_{0,\text{III}}\left(u\right)$	277	306	355	278	277	306	359	276
$TV_{0,\text{IV}}\left(u\right)$	267	289	351	267	272	298	354	272
$TV_{0,V}\left(u\right)$	110	120	90	110	139	152	106	139
$EC_{I-\text{IV}}\left(P_{H}\right)$	2.679	2.740	2.913	2.679	2.680	2.741	2.913	2.680
$EC_{V}\left(P_{H}\right)$	0.370	0.371	0.371	0.371	0.370	0.369	0.371	0.370
$\Delta y_{\max,I}$	3.79	4.29	2.70	3.79	2.11	2.80	1.91	2.11
$\Delta y_{\max,\text{II}}$	3.86	4.36	2.57	3.86	2.08	2.77	1.88	2.08
$\Delta y_{\max,\text{III}}$	0.62	0.72	0.72	0.62	0.72	0.82	0.82	0.72
$\Delta y_{\max,\text{IV}}$	0.69	0.69	0.69	0.69	0.69	0.79	0.79	0.69
$\Delta y_{\max,V}$	0.99	0.89	0.89	0.99	0.99	0.99	0.79	0.99

## Appendix B

Consider the difference output representation (8) and

Δu·s(t):=u·(t)−u·s,ΔPHs(t):=PH(t)−PHs

for the operation point (14), and introduce the notation

Δu(t):=(ΔuPs(t),ΔuCs(t),ΔPHs(t))T,Δϑ(t):=(ΔϑHOs(t),ΔϑCIs(t),ΔϑCOs(t))T

Let ϑa(t)=ϑa=ϑas=24=const., which i.a. means that the ambient temperature is considered to be a model parameter with the given nominal value, not a disturbance input. This option is further used when performing robustness tests.

Apply the simple linearization rule

$$\frac{d}{dt}\Delta \vartheta_{j}\left(t\right)\approx \sum_{i=1}^{3}\frac{\partial }{\partial u_{i}\left(t\right)}{\frac{du_{i}\left(t\right)}{dt}|}_{\Omega }\Delta u_{i}\left(t\right),j=\bar{1,3}$$

for DDEs (1), (3), and (4) after substituting the algebraic (2), (5), (6), and the delayed relation (7), where $\Delta \vartheta_{j}\left(t\right)$ stands for entries of Δϑ(t) and $\Delta u_{i}\left(t\right)$ that of Δu(t). Symbol Ω represents the operating point (14). Then, the following linearized state-space model is received [26]

$$\frac{d}{dt}\Delta \vartheta \left(t\right)=A_{0}\Delta \vartheta \left(t\right)+A_{H}\Delta \vartheta \left(t-\tau_{H}\right)+A_{C}\Delta \vartheta \left(t-\tau_{C}\right)+A_{\text{CH}}\Delta \vartheta \left(t-\tau_{\text{CH}}-\tau_{H}\right)+B_{0}\Delta u\left(t\right)+B_{H}\Delta u\left(t-0.5\tau_{H}\right)+B_{\text{FC}}\Delta u\left(t-\tau_{\text{FC}}\right)$$

in which the matrices read

$$\begin{array}{l} A_{0}=\left(\begin{array}{ccc} A_{1} & 0 & 0\\ 0 & A_{2} & 0\\ 0 & 0 & A_{3} \end{array}\right),A_{H}=\left(\begin{array}{ccc} 0 & 0 & 0\\ A_{4} & 0 & 0\\ 0 & 0 & 0 \end{array}\right),A_{C}=\left(\begin{array}{ccc} 0 & 0 & 0\\ 0 & 0 & 0\\ 0 & A_{5} & 0 \end{array}\right),A_{\text{CH}}=\left(\begin{array}{ccc} 0 & 0 & A_{6}\\ 0 & 0 & 0\\ 0 & 0 & 0 \end{array}\right)\text{,}\\ B_{0}=\left(\begin{array}{ccc} B_{1} & 0 & B_{2}\\ B_{3} & 0 & 0\\ B_{4} & 0 & 0 \end{array}\right),B_{H}=\left(\begin{array}{ccc} 0 & 0 & B_{5}\\ 0 & 0 & 0\\ 0 & 0 & 0 \end{array}\right),B_{\text{FC}}=\left(\begin{array}{ccc} 0 & 0 & 0\\ 0 & 0 & 0\\ 0 & B_{6} & 0 \end{array}\right) \end{array}$$

where thwith static gains adjustmente entries are

A1=−1MH[π0(uPs+π1)π2+h1π02(uPs+π1)2π2+π2π0(uPs+π1)π2PHs+h0(PHs)2+h32c(h5π0(uPs+π1)π2+h4PHs)2],A2=−1cMP[cπ0(uPs+π1)π2+0.5kP],A3=−1cMP[2cπ0(uPs+π1)π2+c2(uCs)2+c1uCs+c0],A4=1cMP[cπ0(uPs+π1)π2−0.5kP],A5=1cMP[2cπ0(uPs+π1)π2−c2(uCs)2+c1uCs+c0],A6=1MH[π0(uPs+π1)π2−h1π02(uPs+π1)2π2+h2π0(uPs+π1)π2PHs+h0(PHs)2+h32c(h5π0(uPs+π1)π2+h4PHs)2],B1=π0π2(uPs+π1)π2−1MH[ϑHIs−ϑHOs+(ϑHIs+ϑHOs2−ϑa)(−h1h5π02(uPs+π1)2π2−2h1h4π0(uPs+π1)π2PHs+(h0h5−h2h4)(PHs)2−h3h5c(h5π0(uPs+π1)π2+h4PHs)2)],B2=ϑHIs−ϑHOs+(ϑHIs+ϑHOs2−ϑa)[(h1h4−h2h5)π02(uPs+π1)2π2−2h0h5π0uPs(uPs+π1)π2PHs−h0h4(PHs)2+h3h4c(h5π0(uPs+π1)π2+h4PHs)2],B3=(ϑHOs−ϑCIs)π2π0(uPs+π1)π2−1MP,B4=(ϑCIs−ϑCOs)π2π0(uPs+π1)π2−1MC,B5=1cMH,B6=−(ϑCIs−ϑCOs2−ϑa)π2π0(uPs+π1)π2−1(2c2uCs+c1)cMC

By using the Laplace transform, the following input-output relation in the *s*-domain via the transfer function matrix can be expressed

(B.1) $$\Delta \vartheta \left(s\right)=G\left(s\right)\Delta u\left(s\right)={\left(sE_{3}-A\left(s\right)\right)}^{-1}B\left(s\right)\Delta u\left(s\right)$$

where

$$A\left(s\right)=\left(\begin{array}{ccc} A_{1} & 0 & A_{6}e^{-\left(\tau_{\text{CH}}+\tau_{H}\right)s}\\ A_{4}e^{-\tau_{H}s} & A_{2} & 0\\ 0 & A_{5}e^{-\tau_{C}s} & A_{3} \end{array}\right),B\left(s\right)=\left(\begin{array}{ccc} B_{1} & 0 & B_{2}+B_{5}e^{-0.5\tau_{H}s}\\ B_{3} & 0 & 0\\ B_{4} & B_{6}e^{-\tau_{\text{FC}}s} & 0 \end{array}\right),E_{33}=\left(\begin{array}{ccc} 1 & 0 & 0\\ 0 & 1 & 0\\ 0 & 0 & 1 \end{array}\right)$$

The reader is referred to Ref. [26] for detailed forms of particular transfer functions in G(s).

## Appendix C

A concise description of the $R_{QM}$ ring follows [49,50].

Definition C.1. A quasi-polynomial is expressed as

$$q\left(s\right)=\left(1+\sum_{j=1}^{k_{n}}q_{nj}e^{-\tau_{nj}s}\right)s^{n}+\sum_{i=0}^{n-1}\sum_{j=0}^{k_{i}}q_{ij}e^{-\tau_{ij}s}s^{i}=q_{n}\left(s\right)s^{n}+\sum_{i=0}^{n-1}\sum_{j=0}^{k_{i}}q_{ij}e^{-\tau_{ij}s}s^{i}$$

where s∈C is the Laplace variable, $q_{ij}\in R$ are coefficients, $0=\tau_{i0}<\tau_{i1}<\ldots <\tau_{ik_{i}}\in R$ mean delays, and $q_{n}\left(s\right)$ is the so-called associated exponential polynomial.

Denote by $r_{Q}\left[s\right]$ the set of quasi-polynomials.

Definition C.2 A fraction $F\left(s\right)=q_{num}\left(s\right)/q_{den}\left(s\right)$ where $q_{num}\left(s\right),q_{den}\left(s\right)\in R_{Q}\left[s\right]$ is formally stable if $q_{n}\left(s\right)$ in $q_{den}\left(s\right)$ has no zero in the left half of the *s*-plane.
Definition C.3 A term $F\left(s\right)\in H_{\infty }$ if ${\left\|F\left(s\right)\right\|}_{\infty }=\underset{Res\geq 0}{\sup}\left|F\left(s\right)\right|<\infty$.
Definition C.4 An element $F\left(s\right)=q_{num}\left(s\right)/q_{den}\left(s\right)\in R_{QM}$, $q_{num}\left(s\right),q_{den}\left(s\right)\in R_{Q}\left[s\right]$, if $q_{num}\left(s\right)=q_{num,0}\left(s\right)e^{-\tau s}$ for some $q_{num,0}\in R_{Q}\left[s\right],\tau \geq 0$, and $F\left(s\right)\in H_{\infty }$ is formally stable.
Definition C.5 $F_{1}\left(s\right)\in R_{QM}$ divides $F_{2}\left(s\right)\in R_{QM}$ if there exists $F_{3}\left(s\right)\in R_{QM}$ so that $F_{2}\left(s\right)=F_{1}\left(s\right)F_{3}\left(s\right)$.
Definition C.6 $F_{1}\left(s\right)\in R_{QM}$ and $F_{2}\left(s\right)\in R_{QM}$ are coprime if there does not exist a noninvertible $F_{3}\left(s\right)\in R_{QM}$ so that $F_{1}\left(s\right)=F_{1,0}\left(s\right)F_{3}\left(s\right)$, $F_{2}\left(s\right)=F_{2,0}\left(s\right)F_{3}\left(s\right)$ for $F_{1,0}\left(s\right),F_{2,0}\left(s\right)\in R_{QM}$.

## Appendix D

Consider the process submodel transfer function (17) representing the model for control purposes using the 1DoF control system. Formulate $G_{m}\left(s\right)$ via $R_{QM}$ according to Definition C.4 as

(D.1) $$G_{m}\left(s\right)=\frac{b\left(s\right)}{a\left(s\right)}=\frac{\frac{b\left(s\right)}{m\left(s\right)}}{\frac{a\left(s\right)}{m\left(s\right)}}=\frac{B\left(s\right)}{A\left(s\right)},A\left(s\right),B\left(s\right)\in R_{QM}$$

where $m\left(s\right)=\left(s+m_{0}\right){\left(s+m_{1}\right)}^{2},m_{0},m_{1}>0$. A possible particular solution of (19) (see Lemma 1) reads (D.2)

(D.2) $$R_{p}\left(s\right)=1\Rightarrow P_{p}\left(s\right)=\frac{1-B\left(s\right)}{A\left(s\right)}=\frac{m\left(s\right)-b\left(s\right)}{a\left(s\right)}$$

Select $Z\left(s\right)\in R_{QM}$ in parameterization (20) as

(D.3) $$Z\left(s\right)=\frac{m\left(s\right)}{a\left(s\right)}\frac{z_{1}s+z_{0}}{s+m_{0}}$$

with some $z_{1},z_{0}\in R$. Then

(D.4) $$\begin{array}{l} P\left(s\right)=\frac{m\left(s\right)-b\left(s\right)}{a\left(s\right)}-\frac{b\left(s\right)}{m\left(s\right)}\frac{m\left(s\right)}{a\left(s\right)}\frac{z_{1}s+z_{0}}{s+m_{0}}=\frac{m\left(s\right)}{a\left(s\right)}-\frac{b\left(s\right)}{a\left(s\right)}\left(1+\frac{z_{1}s+z_{0}}{s+m_{0}}\right)=\frac{m\left(s\right)\left(s+m_{0}\right)-b\left(s\right)\left[\left(1+z_{1}\right)s+m_{0}+z_{0}\right]}{a\left(s\right)\left(s+m_{0}\right)}\\ =\frac{p_{num}\left(s\right)}{p_{den}\left(s\right)} \end{array}$$

Assume a linear-wise r(t) and a step-wise d(t), which means

(D.5) $$F_{r}\left(s\right)=\frac{s^{2}}{f_{r,den}\left(s\right)},F_{d}\left(s\right)=\frac{s}{f_{d,den}\left(s\right)}$$

with some second-order and first-order quasi-polynomials $f_{r,den}\left(s\right)$ and $f_{d,den}\left(s\right)$, respectively, having no zero in the right half-plane. Then, Lemmas 2 and 3 are satisfied simultaneously if

(D.6) $${\left[p_{num}\left(s\right)\right]}_{s=0}=0,{\left[\frac{d}{ds}p_{num}\left(s\right)\right]}_{s=0}=0$$

Conditions (D.6) yield, respectively

(D.7) $$m_{0}^{2}m_{1}^{2}-\left(b_{0}+b_{0D}\right)\left(m_{0}+z_{0}\right)=0\Rightarrow z_{0}=m_{0}\left(\frac{m_{0}m_{1}^{2}}{b_{0}+b_{0D}}-1\right)$$

and

(D.8) $$\begin{array}{l} \frac{d}{ds}p_{num}\left(s\right)=2\left(s+m_{0}\right)\left(s+m_{1}\right)\left(2s+m_{0}+m_{1}\right)+b\left(s\right)\left[\tau_{b}\left(1+z_{1}\right)s-1-z_{1}+\tau_{b}\left(m_{0}+z_{0}\right)\right]+b_{0D}\tau_{0}e^{-\left(\tau_{b}+\tau_{0}\right)s}\left[\left(1+z_{1}\right)s+m_{0}+z_{0}\right]\\ \Rightarrow z_{1}=\frac{2\left(m_{0}^{2}m_{1}+m_{0}m_{1}^{2}\right)-b_{0}-b_{0D}+\left(m_{0}+z_{0}\right)\left[\tau_{b}\left(b_{0}+b_{0D}\right)+\tau_{0}b_{0D}\right]}{b_{0}+b_{0D}} \end{array}$$

By substituting (D.7) into (D.8), and then by substituting (D.7) and (D.8) into (D.4), we obtain

(D.9) $$P\left(s\right)=\frac{p_{num,4}s^{4}+p_{num,3}s^{3}+p_{num,2}s^{2}+p_{num,1}\left(s\right)s+p_{num,0}\left(s\right)}{{\left(b_{0}+b_{0D}\right)}^{2}a\left(s\right)\left(s+m_{0}\right)}$$

the numerator of which is given by (24). According to (20), R(s) then reads (D.10)

(D.10) $$\begin{array}{l} R\left(s\right)=R_{p}\left(s\right)+A\left(s\right)Z\left(s\right)=\frac{m_{0}m_{1}\left(r_{1}s+r_{0}\right)}{\left(s+m_{0}\right){\left(b_{0}+b_{0D}\right)}^{2}}\text{,}\\ r_{1}=2\left(m_{0}+m_{1}\right)\left(b_{0}+b_{0D}\right)+m_{0}m_{1}\left[b_{0}\tau_{b}+b_{0D}\left(\tau_{b}+\tau_{0}\right)\right],r_{0}=m_{0}m_{1}\left(b_{0}+b_{0D}\right) \end{array}$$

Finally, the controller transfer function (23)–(24) reveals from $C_{R}\left(s\right)=R\left(s\right)/P\left(s\right)$.

## Appendix E

Following steps of Appendix D analogously, let $m\left(s\right)={\left(s+m_{0}\right)}^{2},m_{0}>0$ be in (D.1). A possible particular solution of (25) (see Lemma 4) is given by (E.1)

(E.1) $$V_{p}\left(s\right)=1\Rightarrow P_{p}\left(s\right)=\frac{1-B\left(s\right)}{A\left(s\right)}=\frac{m\left(s\right)-b\left(s\right)}{a\left(s\right)}$$

Select $Z\left(s\right)\in R_{QM}$ as in (D.3), which yields P(s) being formally identical to (D.4). Assume (D.5) again. Then the disturbance rejection condition (29) in Lemma 6 requires (D.6) that results in

(E.2) $$m_{0}^{4}-\left(b_{0}+b_{0D}\right)\left(m_{0}+z_{0}\right)=0\Rightarrow z_{0}=m_{0}\left(\frac{m_{0}^{3}}{b_{0}+b_{0D}}-1\right)$$

and

(E.3) $$\begin{array}{l} \frac{d}{ds}p_{num}\left(s\right)=4{\left(s+m_{0}\right)}^{3}+b\left(s\right)\left[\tau_{b}\left(1+z_{1}\right)s-1-z_{1}+\tau_{b}\left(m_{0}+z_{0}\right)\right]+b_{0D}\tau_{0}e^{-\left(\tau_{b}+\tau_{0}\right)s}\left[\left(1+z_{1}\right)s+m_{0}+z_{0}\right]\\ \Rightarrow z_{1}=\frac{4m_{0}^{3}-b_{0}-b_{0D}+\left(m_{0}+z_{0}\right)\left[\tau_{b}\left(b_{0}+b_{0D}\right)+\tau_{0}b_{0D}\right]}{b_{0}+b_{0D}} \end{array}$$

By substituting (E.2) into (E.3), and then by substituting (E.2) and (E.3) into (D.4), it is obtained the form (D.9) of P(s), the numerator of which is given by (31).

The last step (which differs from the 1DoF design) is the reference tracking as per Lemma 5. Parameterization (27) results in (E.4)

(E.4) $$\begin{array}{l} V\left(s\right)=V_{p}\left(s\right)+A\left(s\right)Z\left(s\right)=\frac{m_{0}^{3}\left(v_{1}s+v_{0}\right)}{\left(s+m_{0}\right){\left(b_{0}+b_{0D}\right)}^{2}}\text{,}\\ v_{1}=4\left(b_{0}+b_{0D}\right)+m_{0}\left[b_{0}\tau_{b}+b_{0D}\left(\tau_{b}+\tau_{0}\right)\right],v_{0}=m_{0}\left(b_{0}+b_{0D}\right) \end{array}$$

This form, however, cannot satisfy condition (28) of Lemma 5. Hence, let us extend V(s)=R(s)+Q(s) as

(E.5) $$\tilde{V}\left(s\right)=V\left(s\right)\frac{s+m_{1}}{s+m_{1}}=\frac{m_{0}^{3}\left[v_{1}s^{2}+\left(v_{1}m_{1}+v_{0}\right)s+m_{1}v_{0}\right]}{{\left(b_{0}+b_{0D}\right)}^{2}\left(s+m_{0}\right)\left(s+m_{1}\right)},m_{1}>0$$

and decompose (E.5) as follows

(E.6) $$Q\left(s\right)=\frac{m_{0}^{3}\lambda v_{1}s^{2}}{{\left(b_{0}+b_{0D}\right)}^{2}\left(s+m_{0}\right)\left(s+m_{1}\right)},R\left(s\right)=\frac{m_{0}^{3}\left[\left(1-\lambda \right)v_{1}s^{2}+\left(v_{1}m_{1}+v_{0}\right)s+m_{1}v_{0}\right]}{{\left(b_{0}+b_{0D}\right)}^{2}\left(s+m_{0}\right)\left(s+m_{1}\right)},\lambda \in (0,1]$$

Now, the reference tracking condition (28) holds due to the double zero numerator root of Q(s).

By combining (D.9), (E.2), (E.3), and (E.6), the eventual controller transfer functions (30), (31) are obtained.

## Appendix G

The Smith-predictor reference-to-output transfer function (i.e., its complementary sensitivity function) reads

(G.1) $$T_{Sm}\left(s\right)=\frac{C_{Sm}\left(s\right)G\left(s\right)}{1+C_{Sm}\left(s\right)G\left(s\right)-C_{Sm}\left(s\right)G_{m}\left(s\right)+C_{Sm}\left(s\right)G_{m0}\left(s\right)}$$

By matching (G.1) with (37), one gets (51) directly. Analogously, a comparison of (G.1) with (43) yields (52).

A sketch of a proof of Theorem 1. It can be deduced from the Laplace transform properties that the output excitation caused by the linear-wise load disturbance asymptotically vanishes if the corresponding transfer function satisfies

(G.2) $${\left[G_{dy}\left(s\right)\right]}_{s=0}=0\;\text{and}\;{\left[\frac{d}{ds}G_{dy}\left(s\right)\right]}_{s=0}=0$$

One can check that for 1DoF, the disturbance-to-output transfer function with controller (23)–(24) has form (G.3)

(G.3) $$G_{dy,1\text{DoF}}\left(s\right)=\frac{G\left(s\right)}{1+C_{R}\left(s\right)G\left(s\right)}$$

satisfy (G.2), see also (D.6)-(D.8). On the contrary, the TFC structure with (G.4)

(G.4) $$G_{dy,\text{TFC}}\left(s\right)=\frac{G\left(s\right)}{1+\left[C_{R}\left(s\right)+C_{Q}\left(s\right)\right]G\left(s\right)}$$

does not satisfy (G.2), which means that it is not possible to reject linear-wise load disturbances with controllers (30)–(31).

Hence, the Smith predictor scheme yields

(G.5) $$G_{dy,Sm}\left(s\right)=\frac{G\left(s\right)}{1+C_{Sm}\left(s\right)G\left(s\right)-C_{Sm}\left(s\right)G_{m}\left(s\right)+C_{Sm}\left(s\right)G_{m0}\left(s\right)}$$

By substituting (51) and (52) with particular controllers (23)–(24) and (30)–(31), respectively, into (G.5), it can be found after some algebraic manipulation that (G.6) holds:

(G.6) $${\left[G_{dy,Sm}\left(s\right)\right]}_{s=0}=0\;\text{and}\;{\left[\frac{d}{ds}G_{dy,Sm}\left(s\right)\right]}_{s=0}=-\tau \frac{b_{0}+b_{0D}}{a_{0}+a_{0D}}=-\tau k\neq 0$$

where k means the model static gain. Therefore, the designed Smith predictor cannot reject the linear-wise disturbance unless the input-output delay is zero.

## Nomenclature

**Symbols**
C: set of complex-valued numbers, vectors or functions
*c*: specific heat capacity of water (J kg^−1^ K^−1^)
d(t): load disturbance
e(t): control error
G(s): controlled plant transfer function
$G_{dy}\left(s\right)$: disturbance-to-output transfer function
$C_{R}\left(s\right)$: outer-feedback controller
$C_{Q}\left(s\right)$: inner-feedback controller
$C_{Sm}\left(s\right)$: Smith-predictor controller
G(s): transfer function matrix
$H_{2}$: Hardy space of holomorphic functions with bounded integral of gain squares in the right-half complex plane
$H_{\infty }$Hardy space of holomorphic functions with bounded gain in the right-half complex planej: Hardy space of holomorphic functions with bounded gain in the right-half complex planejimaginary unit (j^2^ = −1)
Im: imaginary part of a complex number or function
**J**: Jacobian matrix
k: heat transmission coefficient (J s^−1^ K^−1^), process static gain
$\dot{m}\left(t\right)$: water mass flow rate (kg s^−1^)
M: water masses (kg)
**p**: static model parameters vector
$P_{H}\left(t\right)$: input heater power (W)
r(t): reference signal
$r_{Q}\left[s\right]$: set of quasi-polynomials
Re: real part of a complex number or function
*R*_*QM*_: ring of quasi-polynomial meromorphic functions
R: set of real-valued numbers, vectors, or functions
*s*: Laplace transform variable
$S_{0}\left(s\right)$: nominal sensitivity function
S(s)perturbed sensitivity function*t*: perturbed sensitivity function*t*time (s)
$T_{0}\left(s\right)$: nominal complementary sensitivity function
u(t): computed control action, manipulated input
$u_{C}\left(t\right)$: voltage input to the heat exchanger fan (V)
$u_{P}\left(t\right)$: voltage input to the pump (V)
$W_{M}\left(s\right)$: uncertainty frequency distribution weight function
$W_{P}\left(s\right)$: frequency distribution sensitivity weight function
y(t): system output
$\Delta y_{\max}$: maximum overshoot/undershoot
${\left\|\cdot \right\|}_{2}$: *H*_2_ norm of a vector or a matrix

**Greek letters**
λ: weight controller parameter
Δ: stead-state deviation, variable change
Δt: sampling period
Δ(s): bounded stable variable function
$\vartheta_{a}\left(t\right)$: ambient temperature (°C)
$\vartheta_{\text{CI}}\left(t\right)$: cooler inlet fluid temperature (°C)
$\vartheta_{\text{CO}}\left(t\right)$: cooler fluid outlet temperature (°C)
$\vartheta_{\text{HO}}\left(t\right)$: heater outlet fluid temperature (°C)
$m_{0},m_{1}$: controller parameters
Ω: operating point
τ: particular delay value (s)

**Subscripts**
*a*: ambient
C: cooler (heat sink, radiator)
*d*: delay
*den*: denominator
F: fan
H: heater
*m*: model
*num*: numerator
O: output
*p*: particular solution
P: piping
*Sm*: Smith predictor
0: nominal system
Superscripts
s: steady state
T: vector or matrix transpose

**Abbreviations**
i.a.: inter alia
DDE: delay differential equation
DPDE: delay partial differential equation
EC: energy consumption
HW: hardware
HX: heat exchanger
IAE: integral absolute error
ODE: ordinary differential equation
PC: personal computer
PDE: partial differential equation
PI: proportional-integral
PID: proportional-integral-derivative
SW: software
TFC: two-feedback-controllers
TTL: transistor-transistor logic
TV: total variation
1DoF: one-degree-of-freedom
